# Quantitative Analysis of Metal-Centered π‑Holes in {TM(cyclen)}^2+^ Complexes

**DOI:** 10.1021/acs.inorgchem.6c01328

**Published:** 2026-05-11

**Authors:** Lucas Gian Fachini, Heloísa de Souza Camilo, Matteo Briganti, Eduardo Lemos de Sá, Giovana Gioppo Nunes

**Affiliations:** † Departamento de Química, 355751 Universidade Federal do Paraná, Centro Politécnico, Jardim das Américas, Curitiba, Paraná 81530-900, Brazil; ‡ Dipartimento di Chimica Ugo Schiff and INSTM RU, 9300 Università deglì Studi di Firenze, Via della Lastruccia 3−13, Sesto Fiorentino, Florence 50019, Italy

## Abstract

Metal-centered π-holes are regions of electronic depletion located above pseudoplanar coordination environments that can promote weak axial interactions. However, their characterization remains limited. In this context, this work establishes quantitative descriptors for identifying and comparing π-holes in transition-metal coordination environments. Here, a high-throughput computational analysis of 1296 {TM­(cyclen)}^2+^ (TM = Co­(II), Ni­(II), Cu­(II), and Zn­(II) and cyclen = 1,4,7,10-tetraazacyclododecane) complexes is presented, considering four macrocyclic conformations and all combinations of methyl/ethyl nitrogen substitutions in cyclen. Electrostatic potentials were evaluated on electron density isosurfaces and analyzed using an algorithm that identifies π-hole candidates. Local maxima along the axis perpendicular to the nitrogen coordination plane are also validated by comparison with surrounding sectors. Validated π-holes, typically located 1.8–2.4 Å above the metal center, were detected in 84.6% of the complexes. Analysis of them shows that the maximum electrostatic potential alone does not reliably describe axial electron depletion in charged complexes. Instead, relative electrostatic descriptors provide consistent measurements of π-hole depth and anisotropy. Across the series examined, the π-hole intensity follows the order Zn­(II) > Co­(II) > Cu­(II) > Ni­(II), in good agreement with experimental findings. Conformational distortions and nitrogen substitution further modulate the magnitude and spatial distribution of the electrostatic potential.

## Graphical abstract

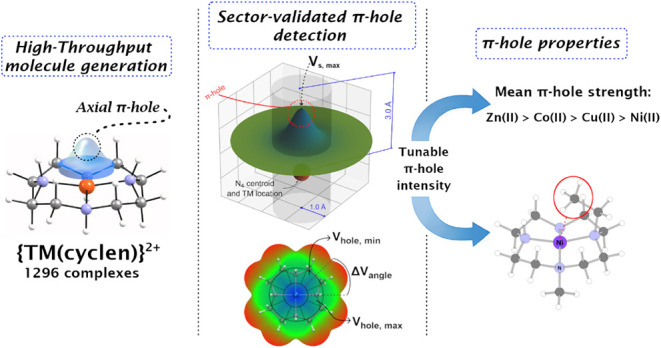

## Introduction

Directional noncovalent interactions arising from the electrostatic potential on molecular surfaces are recognized as design elements in crystal engineering, host–guest chemistry, catalysis, and materials science.^1–4^ In particular, σ-holes and π-holes describe anisotropic electron-depletion zones that enable electrophilic interactions along, or perpendicular to, a bonding direction, respectively. These concepts have been consolidated by electrostatic potential theory and extensively reviewed, establishing a rigorous basis for mapping and quantifying such sites.^5–9^ While σ-hole bonding is ubiquitous in halogen, chalcogen, pnictogen, and tetrel chemistry,^10–12^ π-holes provide complementary directionality above metals in pseudoplanar coordination environments.^5,13^ Notably, metalloporphyrin compounds have been shown to exhibit Br···M contacts that align with π-hole engagement, demonstrating that these interactions are chemically accessible, giving rise to weak yet directional bonds.^14^ Concurrently, semicoordination has become a unifying term for describing low covalence bonds that are not classified as traditional coordination bonds, often stabilized by complementary noncovalent interactions.^15–17^ Experimental and computational investigations into Co­(II), Cu­(II), and Group-10 systems indicate that semicoordination often correlates with π-hole features and can serve as a design element that stabilizes geometries and directs supramolecular organization.^16,18,19^ Semicoordination was also shown to contribute to the thermal and structural stability of Co­(II) complexes with Schiff base ligands, making them suitable for deposition on graphene surfaces.^19^

Electrostatic surface potential (ESP) mapping on isodensity surfaces is a broadly used strategy for describing molecular structure, reactivity, bonding, and recently, σ- and π-holes in a qualitative approach.^20–22^ Attention has increasingly been focused on transition-metal participation in hole-driven noncovalent chemistry since metals can introduce strongly polarized potentials that can either compete with, or reinforce, ligand-localized features, giving rise to coordinate and semicoordinate interactions.^13,14,23,24^ Multiple studies approach how electrostatics, polarization, charge transfer, and dispersion forces collectively influence the energetics of σ- and π-hole interactions in metal complexes.^15,16,25–27^ Scheiner and coworkers have provided analyses of σ- and π-hole behavior, including situations where both operate on the same electrophilic site, as well as the influence of metal ions and other tunable parameters on the interaction intensity.^5,28^ In this context, accurate identification and characterization of π-holes in ESPs is a necessary step to establish a foundation for extending hole-driven interaction toward advancing transition-metal supramolecular chemistry.

Regarding macrocyclic ligands, most existing studies focus on porphyrinoids or on isolated systems, often lacking systematic exploration of the full combinatorial space required to establish general design rules for π-holes and, potentially, semicoordination prediction.^14^ Within this context, 1,4,7,10-tetraazacyclododecane (cyclen) provides an ideal platform to study metal-centered electron anisotropy. As a foundational scaffold in coordination chemistry, cyclen acts as a tetradentate chelator that stabilizes a broad range of metal ions through preorganized N-donor coordination. Extensive studies of cyclen and its derivatives demonstrate that its four nitrogen donors enforce well-defined coordination geometries, while macrocycle functionalization modulates complex stability and selectivity.^29,30^ Decades of tetraazamacrocycle literature further show how conformation, ring substitution, counterions, and secondary interactions govern geometry, lability, and reactivity in Co­(II), Ni­(II), Cu­(II), and Zn­(II) complexes.^31–37^ Collectively, these features make cyclen particularly suited for systematic analysis of metals, substituent and distortion effects on π-holes.

The present work explores this potential using an automated pipeline that generates all four unique conformations of {TM­(cyclen)}^2+^, combinates all the possible methyl and/or ethyl nitrogen substitution patterns and spans the divalent 3d series from Ni­(II) to Zn­(II). The divalent first-row transition metals were selected to extend a previous work involving {TM­(cyclen)}^2+^, in which TM = Ni­(II), Cu­(II), and Zn­(II), complexes and the polyoxometalate [V_10_O_28_]^6–^ named decavanadate or simply V_10_. In that study, the units containing Zn­(II) and Cu­(II) bind to the decavanadate, through a hindered triply bridging (μ_3_-O) oxygen atom in an unprecedented coordination mode, while the Ni­(II) analogue acts as an ionic pair.^38^ Herein, semiempirical preoptimizations and DFT optimizations are conducted for the 1944 resulting molecules, ESP surfaces are computed and analyzed with a novel approach that identify π-hole candidates along the vector normal to the nitrogens plane, then validates each candidate against eight sector local backgrounds within a 2.5 Å radius. This strategy automates ESP analysis, π-hole detection and π-hole intensity calculation in approximately planar transition-metal systems.

This study pursues three primary objectives. First, to establish a quantitative framework for detecting and characterizing metal-centered π-holes in transition-metal complexes using relative electrostatic descriptors derived from sector-based analysis. Second, to determine how metal identity, macrocyclic conformation, and N-substitution patterns control the occurrence and magnitude of π-holes across the {TM­(cyclen)}^2+^ chemical space. Third, to rationalize these trends through correlations with geometric and electronic-structure descriptors, including TM–N distances, τ_4_ distortion, and metal-orbital occupation that govern axial electron depletion.

## Computational Methods

### Overview

To evaluate π-hole formation across the {TM­(cyclen)}^2+^ chemical space, an automated workflow was developed to generate, optimize, and analyze several hundred structures varying in metal center (TM = Co­(II), Ni­(II), Cu­(II), and Zn­(II)), macrocyclic conformation, and N-substitution pattern (hydrogen, methyl or ethyl). The overall workflow is summarized in Figure 1.

**Figure 1:**
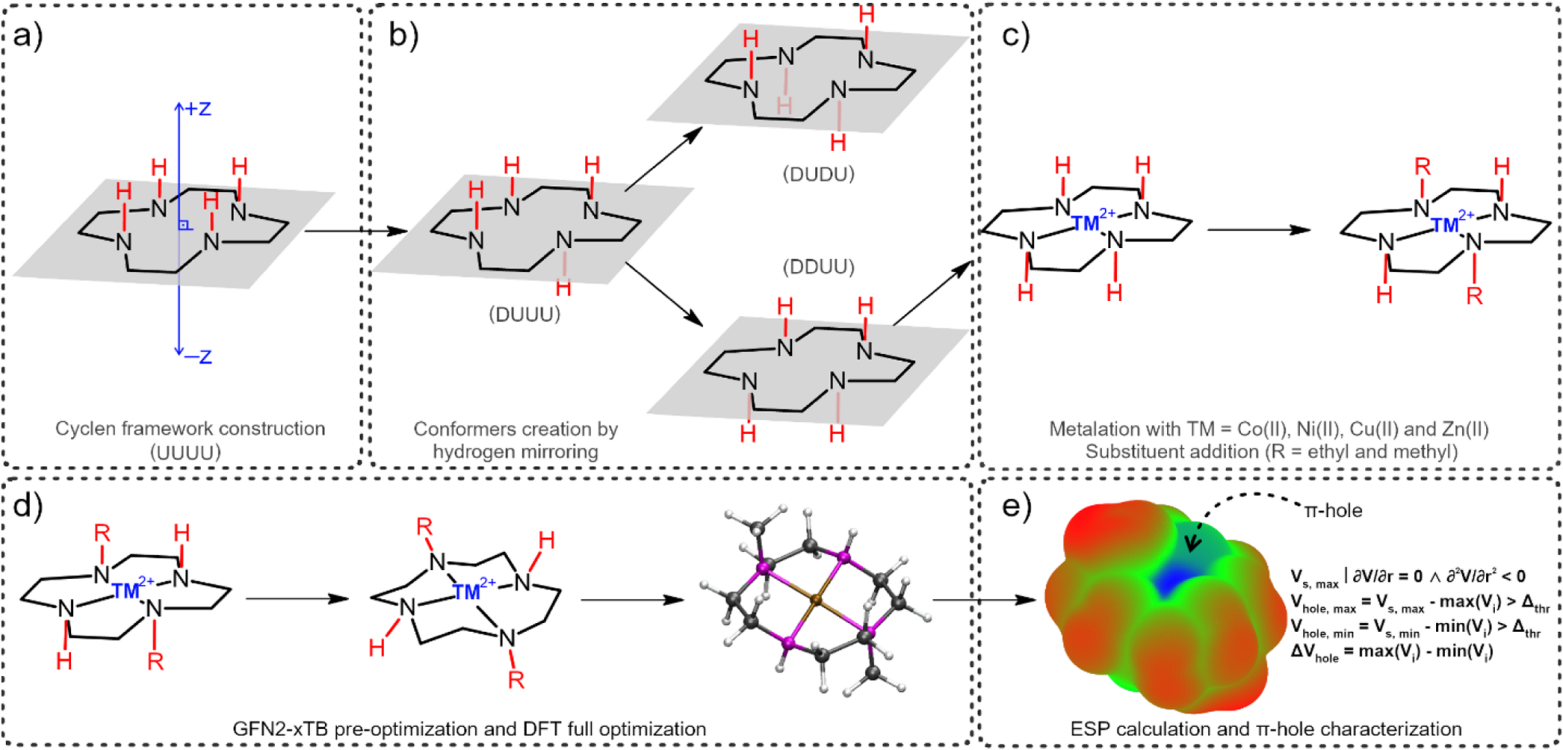
Computational workflow used to generate and analyze the {TM­(cyclen)}^2+^ data set. (a) Construction of the cyclen framework and definition of the N_4_-plane. (b) Generation of the four cyclen conformers (UUUU, DUUU, DDUU, DUDU) by mirroring N–H orientations. (c) Metalation with TM = Co­(II), Ni­(II), Cu­(II), and Zn­(II) followed by combinatorial N-substitution (R = methyl or ethyl). (d) GFN2-xTB preoptimization and DFT geometry refinement. (e) Electrostatic potential (ESP) calculation and automated π-hole identification and quantification.

To implement the workflow for high-throughput calculations, a cyclen template was first created for each unique conformer labeled as UUUU, DUUU, DUDU, and DDUU (where U and D denote the up and down orientations of the N-substituents relative to the macrocyclic plane) (Figure 1a and b). The desired TM cations were then added in a combinatorial manner to the cyclen template, followed by substitution of the N-bound hydrogens with the selected R groups (R = methyl or ethyl, Figure 1c). Atom-overlap testing was incorporated to eliminate unrealistic structures, subsequently, the remaining molecules were preoptimized at a semiempirical quantum-mechanical level using the GFN2-xTB method^39^ aiming to accelerate the final DFT optimization. The DFT-generated wave functions were used to calculate ESP maps using the Multiwfn (v3.8) in batch mode (Figure 1d).^40^ Finally, an algorithm was applied to detect π-hole candidates on each calculated ESP, employing a gradient search over the molecular plane. The electrostatic potential of π-hole candidates was compared with ESP values in the surrounding region, since a π-hole corresponds to a locally enhanced electrostatic potential relative to its neighborhood, represented by the blue regions in Figure 1e.^5^

### Molecular Generation and Conformational Space

The cyclen molecule can adopt several protonation states, ranging from the neutral cyclen to the fully protonated cyclenH_4_^4+^.^41^ In the present study, the nonprotonated form was selected for investigation. This species exhibits a rich conformational space when coordinated to TM, as all four nitrogen atoms bind to the metal center while the remaining N–H bonds can be oriented either above or below the plane containing the four nitrogen atoms (N_4_-plane).^42–44^ Initially, a planar backbone was constructed for the cyclen ligand with all four N–H groups oriented in the same direction (UUUU, Figure 1a). From this reference structure, the corresponding hydrogens were mirrored across the N_4_-plane to generate the other three unique cyclen conformations, denoted DUUU, DUDU and DDUU. As additional reference, U indicates an N–H vector pointing along the positive *z*-axis and D corresponds to one directed along the negative *z*-axis (Figures 1a and S1). Each conformer was then combined with a TM (Co­(II), Ni­(II), Cu­(II), and Zn­(II)) placed at the centroid of the four nitrogen atoms (Figure 1c), affording a set of 16 initial structures.

After building the {TM­(cyclen)}^2+^ starting set, a combinatorial N-substitution step was performed to replace hydrogens by methyl and/or ethyl groups, in every possible combination, aiming to verify their impact into π-hole. This procedure generated 1280 substituted structures in addition to the 16 unsubstituted ones from the initial set, for a total of 1296 molecules in the data set. To perform the substitution, a straightforward geometric approach was employed (Figure 2). For each N–H bond, a reference vector was defined, the hydrogen atom was removed, and the R group was placed along the reference vector at a fixed N–C distance of 1.47 Å, employing a dummy atom, X, to define the bond orientation. After each R group placement, all interatomic distances were computed; any value shorter than 0.8 Å flagged the structure as unphysical. In such cases, the R group was rotated around the reference vector in 10° increments until no atomic overlap was detected. Once the structure passed this overlap check, it was stored for subsequent optimization steps. A threshold of 0.8 Å was chosen for overlap detection because it is significantly shorter than a C–H bond,^45^ the shortest bond length expected in this type of system.

**Figure 2:**
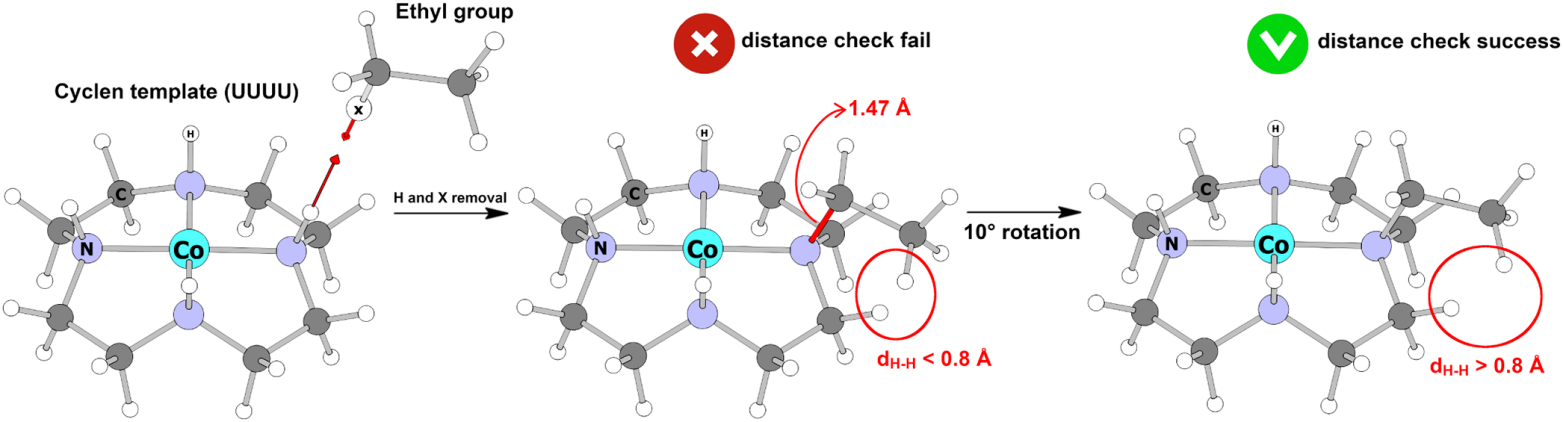
Procedure used for N-substitution in the {TM­(cyclen)}^2+^ structures. A hydrogen atom bound to nitrogen is removed and replaced by an ethyl group positioned along the original N–H vector at a fixed N–C distance of 1.47 Å. Interatomic distances are then checked to detect steric overlaps; structures presenting contacts shorter than 0.8 Å are rejected and the substituent is rotated in 10° increments until a valid geometry without atomic overlap is obtained.

The molecular generation procedure successfully produced all 1296 expected structures. To verify structural uniqueness, a root mean squared deviation (RMSD) filter with a threshold of 1.5 Å, was applied to identify possible redundancies among structures with identical atom counts. All systems were confirmed to be unique, consistent with the combinatorial design.

### Quantum Chemical Calculations and ESP Evaluation

Each structure was subjected to a GFN2-xTB^39^ and a full DFT optimizations at the ωB97X-D3/def2-SVP level^46–48^ using the RIJCOSX approximation^49^ as implemented in ORCA (v6.0.1).^50,51^ For simplicity, the lowest possible spin multiplicity was employed in all calculations. All other parameters were left at their default values to maintain a streamlined computational protocol. Notably, the inclusion of semiempirical preoptimization eliminated most common job failures, whereas direct DFT optimizations frequently suffered from SCF convergence issues.

ESP analysis was employed to characterize anisotropic charge distribution in each optimized complex and to locate potential π-hole regions. All calculations were conducted on electron-density isosurfaces at ρ = 0.001 bohr^–3^, a threshold commonly adopted for visualizing molecular ESPs and ensuring comparability across systems.^52^ The ESP values were computed on a uniform three-dimensional grid (0.25 bohr) generated employing wave functions obtained at the ωB97X-D3/def2-SVP theory level through the Multiwfn (v3.8) software.^40^

To enable direct comparison of ESP patterns across different conformers and substitution patterns, all maps were transformed into a common molecular reference frame (aligned frame) (Figure S2). The four cyclen nitrogen atoms define the N_4_-plane, whose geometric centroid was used as the origin of the coordinate system. A least-squares fitting procedure was applied to the nitrogen positions to determine the best-fit plane and its normal vector, *n̂*, which serves as the molecular pseudo-*z*-axis (π-axis). The *x*- and *y*-axes were defined within the N_4_-plane using the two shortest noncollinear N–N vectors, ensuring consistent orientation among conformers regardless of distortion. Each ESP grid was then translated and rotated into this aligned frame according to

$$r_{\mathrm{aligned}}=\left(r_{\mathrm{original}}-r_{\mathrm{centroid}}\right)\mathrm{Rot}$$

where **Rot** is the rotation matrix that maps the original coordinates onto the reference frame and r are coordinates. This standardization is essential for the consistent identification and comparison of π-hole features across all 1296 complexes.

### Automated π-Hole Detection Algorithm

An automated algorithm was developed to identify and validate π-hole regions on the ESP surfaces of {TM­(cyclen)}^2+^ complexes. This procedure converts the raw ESP grid data into quantitative descriptors of π-hole intensity and location, ensuring that all detections are chemically meaningful and directly comparable across metals, conformers, and substitution patterns. The methodology described herein is generalizable to other planar or quasi-planar metal systems.

Following coordinate alignment, the ESP was systematically sampled along the positive (+*z*) and negative (−*z*) directions, corresponding to the regions above and below the N_4_-plane, respectively. Each sampling path originated at the N_4_-plane centroid and extended up to 3.0 Å along the *z*-axis within a cylindrical region of radius 1.0 Å, thereby encompassing the spatial domain where charge depletion may give rise to electrophilic sites. Within each cylinder, a gradient-based search was performed to locate local ESP maxima. Points that satisfied both first- and second-derivative criteria (∂V/∂r = 0 and ∂^2^V/∂r^2^ < 0, in which V is the electrostatic potential) were flagged as π-hole candidates (Figure 3a). Each candidate was characterized by its perpendicular distance from the N_4_-plane, ESP magnitude, and curvature (second derivative value), providing quantitative measures of the corresponding electron-depleted region.

**Figure 3:**
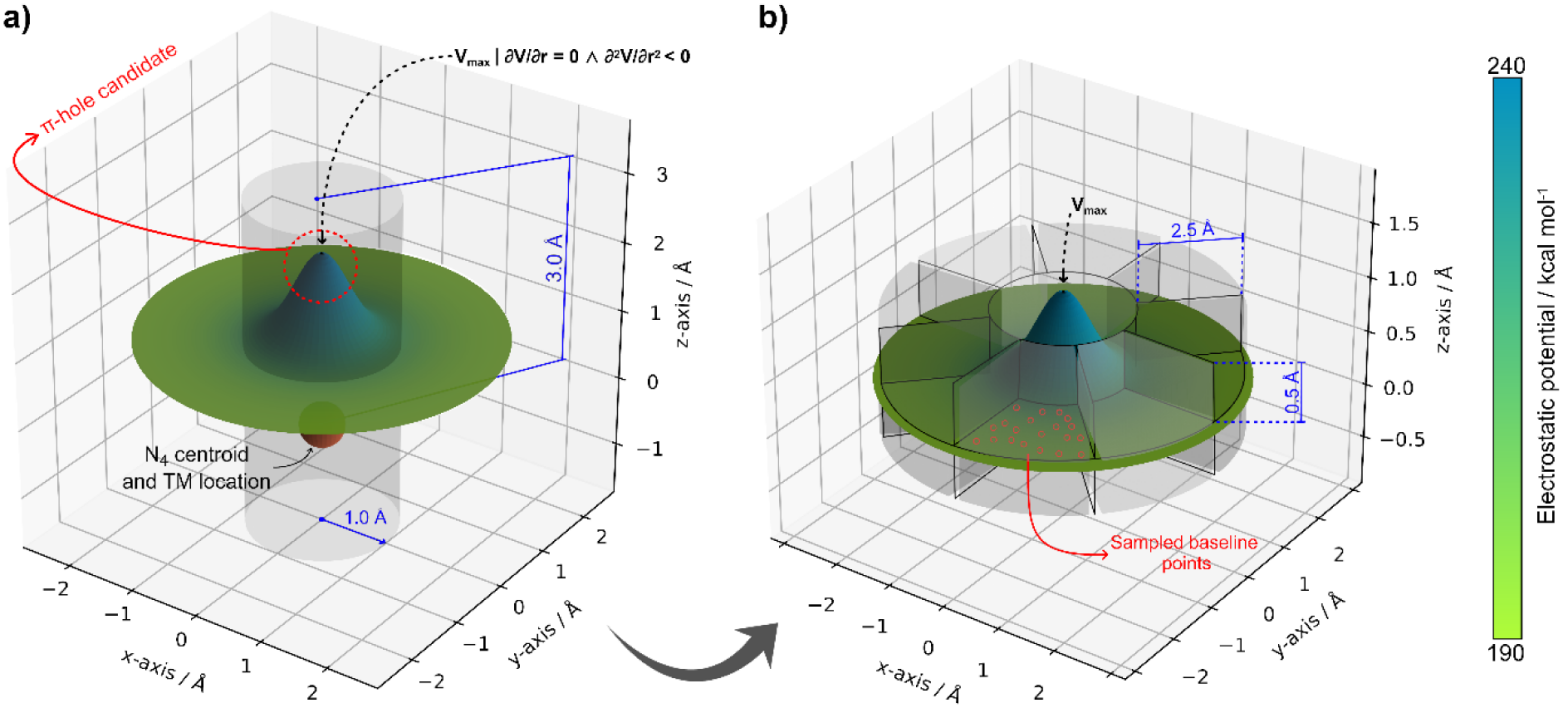
(a) Identification of π-hole candidates by searching for local maxima of the electrostatic potential along the axis perpendicular to the N_4_-plane within a cylindrical region (radius 1.0 Å, height 3.0 Å) centered at the metal atom. (b) Sector-based validation of the candidate π-hole, where the surrounding region (radius 2.5 Å, height ±0.25 Å) is divided into eight sectors to sample baseline ESP values and confirm that the detected maximum corresponds to a genuine region of locally enhanced electrostatic potential.

To discriminate genuine π-holes from random fluctuations, a sectoral validation scheme was implemented. Around each candidate point, a cylindrical region of radius 2.5 Å and height ±0.25 Å was constructed and divided into eight azimuthal sectors (45° each) within the *xy*-plane. For every sector, 1000 evenly distributed ESP samples were collected. The mean sectoral potential *V̅_i_* served as a local background reference. A candidate was retained as a true π-hole only if:

$$V_{hole,mean}=V_{s,max}-mean\left({\mathrm{V̅}}_{i}\right)>\Delta_{\mathrm{thr}}$$

where Δ_thr_ = 5 kcal mol^–1^, a threshold chosen to exclude minor surface undulations and to retain only π-holes of chemical significance. This criterion ensures that each detected maximum corresponds to a region of positive electrostatic potential relative to its local environment. Another pair of useful descriptors are the *V*_*hole,max*_ and the *V*_*hole,min*_ values, defined as the potential difference between the π-hole and the sector exhibiting the lowest or highest mean ESP, respectively. *V*_*hole,max*_ provides a practical metric for comparing the maximum relative depth of electron depletion and the *V*_*hole, min*_ quantifies its minimum counterpart (Figure 4a). Taken together, these descriptors yield: Δ*V_hole_* = *V_hole_*,*_max_* – *V_hole,min_* = *max*(*V̅_i_*) – *min*(*V̅_i_*), a measure of the asymmetry in electron depletion around the π-hole (Figure 4a and b). Since absolute ESP magnitudes vary across metals, oxidation states and ligand environments, the *V*_*hole*_ descriptors serve as a consistent, system-independent measurement of π-hole intensity and asymmetry.

**Figure 4:**
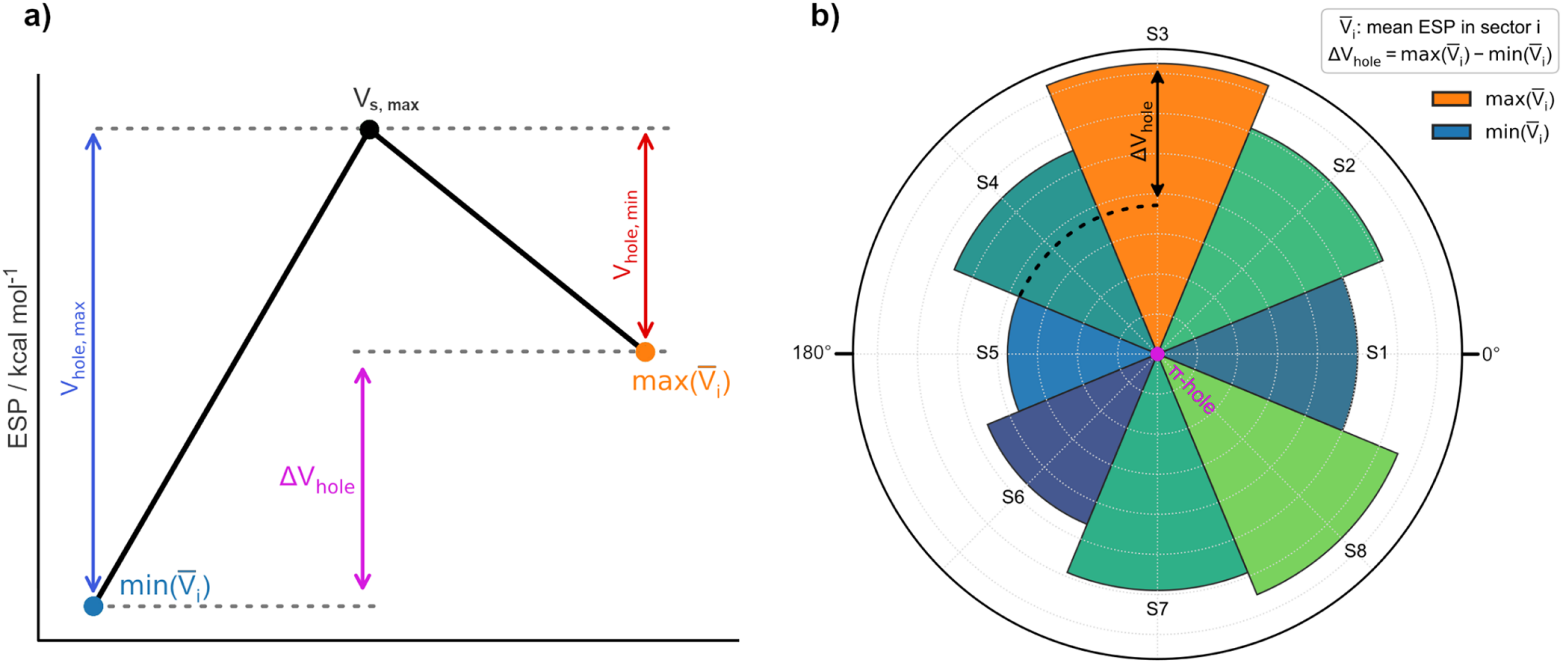
Graphical definition of the descriptors used to quantify π-hole intensity and anisotropy. (a) Schematic representation of the electrostatic potential relationships used to define *V*_*hole,max*_, *V*_*hole,min*_, and Δ*V*_*hole*_ from the sectoral mean potentials *V̅_i_*. (b) Polar representation of the sector-based analysis around the π-hole, where the surrounding region is divided into eight sectors (S1–S8) and the mean ESP of each sector is used to evaluate the magnitude and angular anisotropy of the π-hole through Δ*V*_*hole*_ = *max*(*V̅_i_*) – *min*(*V̅_i_*).

### Data Set Validation, Grid Sensitivity and Data Deposition

All steps, from ESP file parsing to candidate validation, were executed automatically via Python scripts interfaced with NumPy, SciPy, and Pandas libraries. Parallelization across CPU cores allowed simultaneous DFT calculations of hundreds of complexes. Each geometry included in the final data set satisfied stringent DFT convergence criteria, with energy changes, gradient norms, and maximum force components well within standard thresholds for ωB97X-D3/def2-SVP optimizations. ESP surfaces were evaluated on ρ = 0.001 au isodensity using Multiwfn 3.8.

To ensure that the π-hole descriptors were not artifacts of the sampling resolution, a grid-sensitivity analysis was performed. A subset of complexes (100 structures) was recomputed by reducing the grid spacing from the default value of 0.25 to 0.20 bohr, corresponding to an almost 2-fold increase in the number of grid points. Comparison of *V*_*s,max*_, *V*_*hole,mean*_, *V*_*hole,max*_ and *V*_*hole,min*_ obtained with the two grid settings revealed mean deviations below 5% (Figures S3 and S4), indicating tolerable grid dependence. The Δ*V*_*hole*_ descriptor exhibited a slightly larger deviation of 5.71%, which remains modest given that this quantity depends simultaneously on *V*_*hole,max*_ and *V*_*hole,min*_ and is therefore intrinsically more sensitive to grid resolution.

A benchmark comparison (Figure S5) shows that including the GFN2-xTB preoptimization step substantially reduces the total computational time required for DFT optimizations, decreasing the average runtime from approximately 15,000–23,000 s to about 2,000–6,000 s depending on the metal center, corresponding to an overall speedup of roughly 1 order of magnitude. Finally, to account for the influence of the basis set on the calculations, the effect of increasing the basis set from def2-SVP to def2-TZVP was evaluated using a test set of 16 unsubstituted molecules encompassing all combinations of metal centers and conformations (Figure S6). The results indicate that, although the absolute values of *V*_*s*,*max*_, *V*_*hole,mean*_, and Δ*V*_*hole*_ are systematically shifted, the qualitative trends are preserved. The overall changes in the electrostatic potential induced by the choice of basis set affect both *V_s_*,_*max*_ and *V*_*hole,mean*_ in a similar manner, with the latter showing lower sensitivity. In contrast, the asymmetry descriptor exhibits a distinct behavior, with more distorted geometries displaying larger deviations. This likely reflects enhanced polarization effects when using the larger basis set, underscoring the importance of basis set selection for quantitative analyses of electrostatic anisotropy.

All raw and processed data generated in this study have been deposited in the NOMAD Repository (The Novel Materials Discovery Laboratory, https://nomad-lab.eu), ensuring compliance with FAIR (Findable, Accessible, Interoperable, and Reusable) data principles. Each entry corresponds to a fully optimized {TM­(cyclen)}^2+^ complex, including geometry (.xyz), and outputs (.out). The data set encompasses all four divalent first-row transition metals (Co–Zn), four cyclen conformations (UUUU, DUUU, DUDU, DDUU), and all possible N-substitution patterns (methyl/ethyl combinations), totaling 1296 unique systems.

The repository is organized hierarchically by metal identity and conformational type to facilitate targeted data retrieval. Metadata automatically parsed by NOMAD include computational method (ωB97X-D3/def2-SVP), total electronic energy, molecular charge and multiplicity. Each entry is individually citable through a persistent Digital Object Identifier (DOI), and the complete data set is accessible under an open Creative Commons Attribution (CC BY 4.0) license.

Processed descriptor tables, containing π-hole metrics and all associated Python scripts for ESP alignment, sectoral validation, and descriptor extraction might be provided on author request. Together, these materials enable full reconstruction of the automated workflow described herein, supporting reuse for benchmarking, comparative electrostatic studies, and future machine-learning model training. Data DOI: https://doi.org/10.17172/nomad.vw6z-b7wn.

## Results and Discussion

### Data Set Overview and Global Features of π-Hole Formation in {TM­(cyclen)}^2+^ Complexes

The employed workflow yielded a data set encompassing 1296 optimized {TM­(cyclen)}^2+^ complexes, spanning four metals (Co­(II), Ni­(II), Cu­(II), Zn­(II)), four conformational motifs (UUUU, DUUU, DUDU, DDUU), and all the unique combinations of *N*-methyl and N-ethyl substitution patterns. Each molecule was successfully preoptimized with the GFN2-xTB method, followed by full DFT refinement and electrostatic analysis under uniform computational conditions.

ESP surfaces were generated and aligned for all optimized structures. The π-hole detection algorithm identified at one validated π-hole in 84.6% of the systems (Figure 5a), while conformational and metal-dependent effects accounted for the remaining cases where π-holes were not detected. These latter structures showed nearly isotropic electrostatic distributions (*V*_*hole*,*mean*_ < 5 kcal mol^–1^), often arising from distorted geometries or from conformations in which hydrogen atoms from ethyl substituents occupy the axial region and suppress π-hole development.

**Figure 5:**
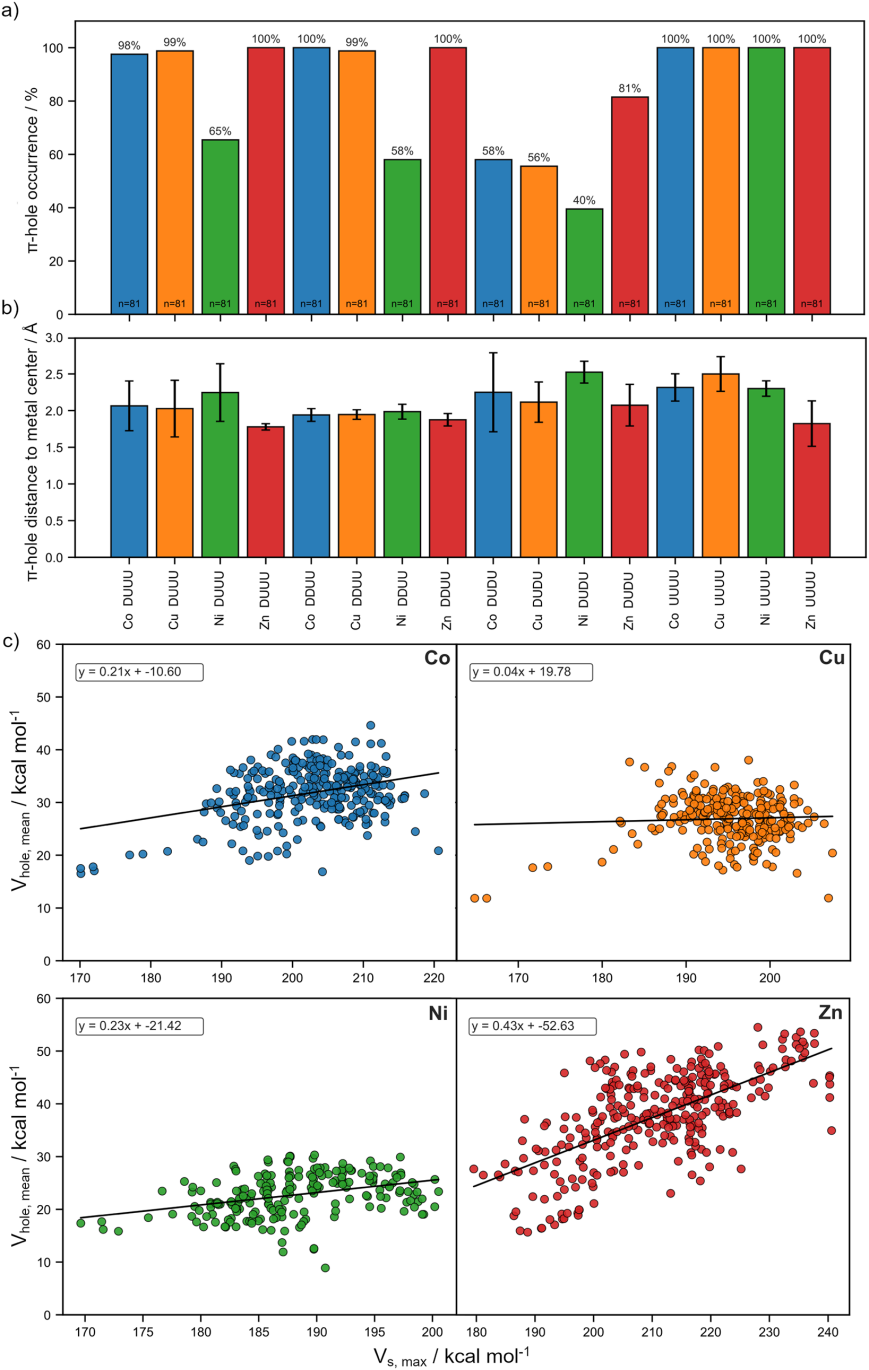
Global characteristics of π-hole in the {TM­(cyclen)}^2+^ data set. (a) Percentage occurrence of validated π-holes for each metal (Co­(II), Cu­(II), Ni­(II), Zn­(II)) and conformational pattern (DUUU, DDUU, UUUU). (b) Average distance between the π-hole position and the metal center for the same metal-conformer combinations; error bars represent standard deviations. (c) Correlation between the maximum electrostatic potential *V_s_*,_*max*_ and the mean π-hole depth *V*_*hole*,*mean*_ for each transition metal, showing the weak dependence between absolute ESP maxima and relative π-hole intensity across the data set.

The results indicate that π-holes are an intrinsic but adjustable feature of the cyclen coordination environment, with axial electron depletion above the N_4_-plane appearing consistently across all transition-metal ions studied. The detected π-holes were localized approximately 1.8–2.4 Å above the metal center (Figure 5b). The lower end of this range is typically associated with bonding interactions of neutral or anionic Lewis bases, while the higher end is often correlated with semicoordination bonds.^15–19^

It is also important to note that the maximum ESP value, *V_s,max_*, by itself, is not a reliable measure of π-hole strength. The *V_s,max_* based criterion performs especially poorly for charged systems: cations can display a uniformly positive ESP without exhibiting any π-hole (*V_s,max_* >0), whereas anions may contain well-defined π-holes despite having a negative surface potential (*Vs,max* < 0). In this context, the plotted *V*_*hole*,*mean*_ against *V_s,max_* (Figure 5c) indicates that the maximum ESP value is not directly related to the depth of electron depletion in {TM­(cyclen)}^2+^ complexes. For Co­(II) and Ni­(II), most structures display *V*_*hole*,*mean*_ values of approximately 30 and 20 kcal mol^–1^, respectively, while their *V_s,max_* values span a broad range from roughly 170 to over 200 kcal mol^–1^. Cu­(II) complexes show a similar pattern, with little apparent connection between the two descriptors. Zn­(II) exhibits a somewhat clearer correspondence between *V_s,max_* and *V*_*hole*,*mean*_, but the large spread still points to a weak correlation. This reinforces the necessity of employing relative measurements of the ESP to identify regions of electron depletion.

### Metal-Dependent and Conformer-Dependent Trends in π-Hole Strength

To better understand which factors influence the π-hole strength a detailed molecular orbital (MO) analysis was conducted for the studied complexes, aiming to correlate *V*_*hole*,*mean*_ values with transition-metal microsymmetry, considering geometric distortions from D_4h_ toward T_d_ or C_4v_. To quantify this distortion along the square-planar to tetrahedral range, the four-coordinate geometry index *τ*_4_ was employed.^53^ This parameter, defined from the two largest L–M–L angles, provides a continuous metric in which *τ*_4_ = 0 corresponds to an ideal square-planar geometry, whereas τ_4_ = 1 represents a tetrahedral arrangement. For reference, the mean *τ*_4_ obtained for each metal-conformer combination explored in this work is shown in Figure S7.

All complexes were initially analyzed in their lowest accessible spin states, adopting the low-spin configuration consistent with a pseudoplanar geometry (Figure 6c). Under these conditions, the crystal-field splitting is expected to follow the approximate ordering $d_{xz},d_{yz}<d_{z^{2}}<d_{xy}<d_{x^{2}-y^{2}}$ , with modest variations depending on the geometry distortion, particularly in the relative separation of $d_{z^{2}}$ and *d_xy_*. Upon combination with the σ-donor orbitals of the cyclen ligand, the resulting molecular orbitals are expected to stabilize the b_1g_, a_1g_, and e_u_ bonding sets. The b_2g_ and e_g_ nonbonding orbitals experience only minor splitting, while the a_1g_ nonbonding level is further destabilized through mixing with the metal 4s orbital (Figure 6a). The b_1g_* antibonding orbital, dominated by $d_{x^{2}-y^{2}}$ character, is the highest-energy d-derived MO in this manifold. Within this framework, the $d_{x^{2}-y^{2}}$ (b_1g_) orbital is aligned directly with the equatorial nitrogen donors, whereas the $d_{z^{2}}$ (a_1g_) orbital, oriented perpendicular to the N_4_-plane, remains comparatively lower in energy.

**Figure 6:**
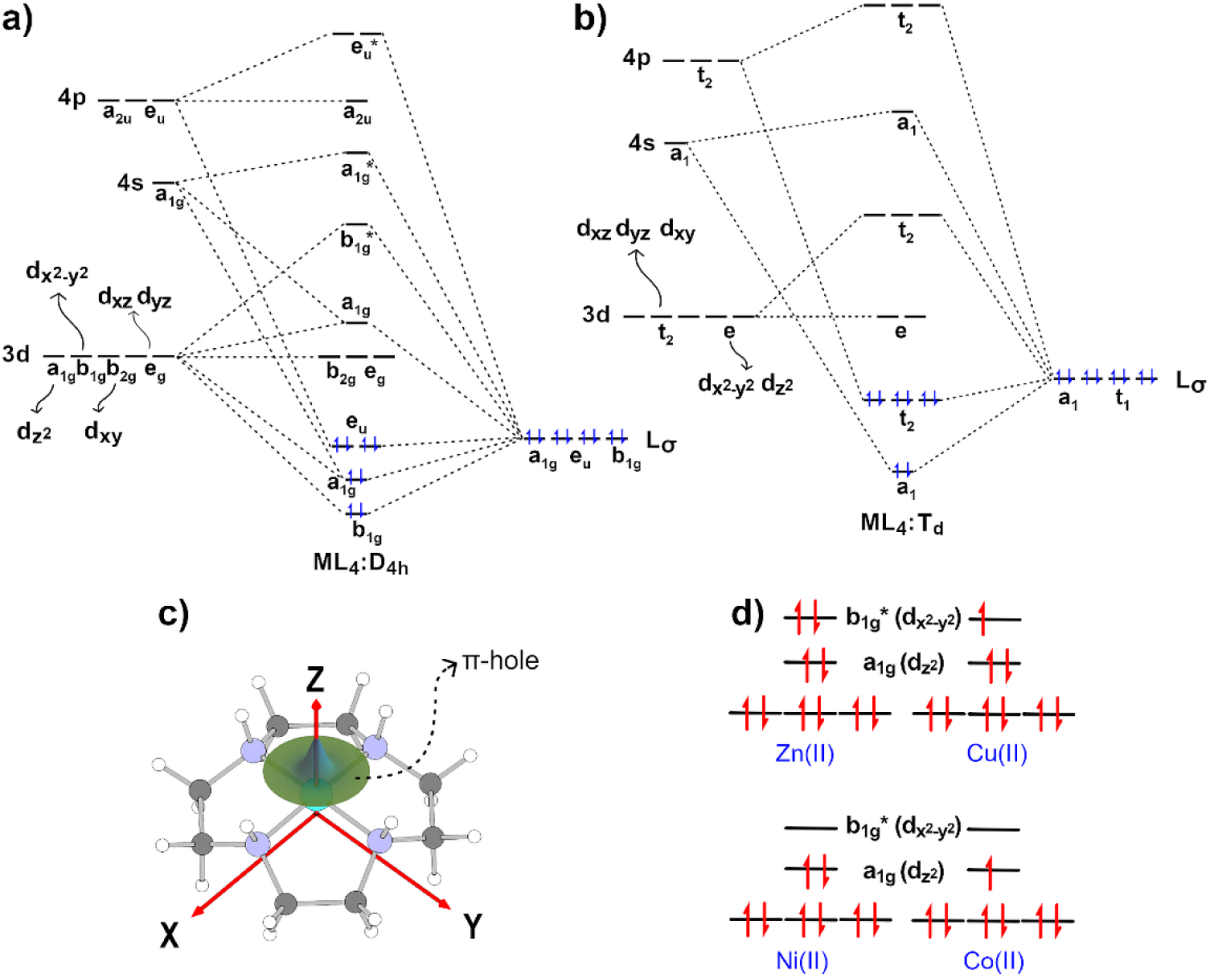
Qualitative molecular orbital diagrams for complexes with σ-bonding-only ligands for a) square-plane and b) tetrahedral geometries. c) Pseudoplanar geometry of the {TM­(cyclen)}^2+^ framework, highlighting the N_4_-plane and the axial (*z*-axis) direction along which the π-hole emerges. d) Approximate d-orbital splitting in a pseudoplanar ligand field.

The observed π-hole intensity sequence Zn­(II) > Co­(II) > Cu­(II) > Ni­(II) (Figure 7) cannot be rationalized by d-shell vacancy alone, as Zn­(II) exhibits the deepest π-holes despite its closed-shell configuration. Moreover, *V_hole_*,*_mean_* does not vary monotonically across the d^8^ → d^10^ progression, indicating that the π-hole strength arises from a more complex interplay of electronic factors. Increasing the b_1g_* antibonding orbital population weakens equatorial σ-donor interactions, reducing electron density donation to the metal center, and thereby, enhancing axial electronic depletion which is manifested as a π-hole. For Cu­(II), in which the b_1g_* level is only partially populated, this effect is less pronounced than for Zn­(II). Ni­(II) complexes, in contrast, do not have populated b_1g_* MO, leading to stronger σ-donation and a reduced tendency to develop π-holes. For Co­(II) complexes, limited to their low-spin d^7^ configuration, the b_1g_* MO is also unpopulated, but, in contrast, have a direct vacancy in the $d_{z^{2}}$ (a_1g_) MO, generating an axial electronic depletion. The combined influence of a_1g_ vacancy and b_1g_* population, therefore, may explain the *V_hole,mean_* ordering observed across the {TM­(cyclen)}^2+^ series.

**Figure 7:**
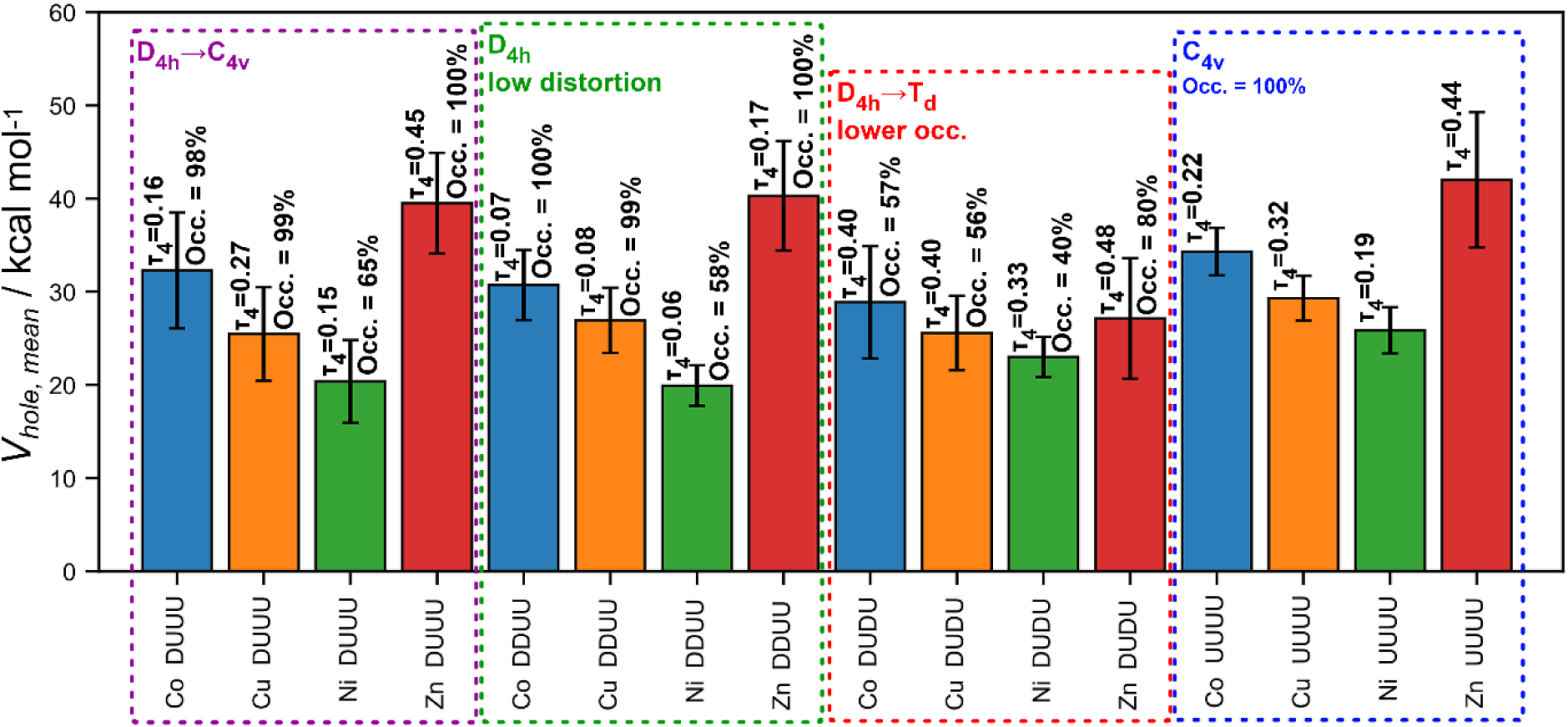
Mean π-hole intensity *V_hole,mean_* for {TM­(cyclen)}^2+^ complexes as a function of metal center and macrocyclic conformation. Bars represent average values for each metal–conformer combination (Co­(II), Cu­(II), Ni­(II), Zn­(II); DUUU, DDUU, UUUU) with standard deviations. The τ_4_ parameter and π-hole occurrence are indicated above each bar. Colored boxes highlight the dominant geometric regimes, illustrating the influence of coordination distortion from pseudoplanar (D_4h_) toward tetrahedral (T_d_) and pyramidal (C_4v_) arrangements on π-hole intensity.

The *V_hole,mean_* ordering also depends on how much the geometry is distorted from a square-planar arrangement (Figure 7). Increasing tetrahedral character generally reduces π-hole intensity, whereas an off-plane displacement to a square- pyramidal coordination geometry (C_4v_) of the metal often enhances the positive ESP region above the molecular plane. Although this trend may appear straightforward, it reflects deeper changes in the orbital composition of the complexes. In a T_d_ environment, the $d_{x^{2}-y^{2}}$ and $d_{z^{2}}$ orbitals are predominantly nonbonding (Figure 6b); therefore, as the ligand field distorts from a D_4h_ to a tetrahedral symmetry, the antibonding contribution of the populated orbitals decreases. Consequently, complexes with strong T_d_ character, such as DUDU Zn­(II) (τ_4_ ≈ 0.49), exhibit a substantially reduced capacity to form π-holes. A similar effect is observed for Cu­(II), where the most tetrahedrally distorted conformer (DUDU) displays fewer and weaker π-holes than their more planar or off-plane-distorted counterparts (UUUU and DDUU). For Co­(II), tetrahedral distortion is pronounced only in the DUDU conformer, with minimal impact in the remaining geometries. In contrast, Ni­(II) is less responsive to orbital changes generated by geometrical distortions. The $d_{x^{2}-y^{2}}$ antibonding orbital is unpopulated in D_4h_-like environments, while in T_d_-like environments this orbital is populated but nonbonding, disfavoring π-holes in both cases. The main effect of the T_d_ distortion in the DUDU conformer (τ_4_ ≈ 0.33) is a reduced occupation of the *d*_*xz*_ and *d_yz_* orbitals, which introduces some directional electron depletion along *z*-axis, but does not increase the π-hole intensity since $d_{z^{2}}$ is populated.

Finally, the UUUU conformer (C_4v_) exhibits a predominantly off-plane distortion that stabilizes the $d_{x^{2}-y^{2}}$ orbital while raising the energy of *d*_*xz*_ and *d_yz_*.^54,55^ As mentioned above, for all metal ions adopting this conformation, the equatorial σ-bonds are weakened, impairing ligand–metal charge transfer, enhancing electron depletion. For instance, σ-bond weakening is clearly observed in N–Zn bond lengths, which increase from ca. 2.01 Å in DUDU to 2.09 Å in UUUU (Figure 9).

### Metal-Dependent π-Hole Strength and Its Impact on Directional Binding

It is noteworthy that the Zn­(II) > Co­(II) > Cu­(II) > Ni­(II) π-hole strength trend identified herein is consistent with previous reports on metalloporphyrin systems,^14^ indicating that π-hole-mediated supramolecular interactions follow a comparable ordering. Furthermore, this trend aligns with prior observations that decavanadate anions preferentially bind to {TM­(cyclen)}^2+^ complexes exhibiting pronounced π-holes (e.g., Zn­(II) and Cu­(II)), whereas such interactions are significantly weaker or absent for Ni­(II).^38^

Revisiting our previous study in which the decavanadate interacts with cyclen complexes containing Cu­(II), Ni­(II) and Zn­(II), π-hole descriptors were calculated for the optimized {TM­(cyclen)}^2+^ moieties using the def2-TZVP basis set, based on geometries derived from crystallographic data of the ionic pair [Ni­(cyclen)­(H_2_O)_2_]_2_[H_2_V_10_O_28_]·2H_2_O and the discrete molecular entities [{Cu­(cyclen)}_2_(H_2_V_10_O_28_)]·9H_2_O, and [{Zn­(cyclen)}_3_(V_10_O_28_)]·4H_2_O (Figure S8). The results shown in Table 1 indicate that {Ni­(cyclen)}^2+^ does not exhibit significant electron depletion over the metal center. Although a local electrostatic potential maximum is identified along the axial direction, it fails the sectoral validation criterion, yielding a negative *V_hole,mean_* value of −0.90 kcal mol^–1^, consistent with the absence of a true π-hole. In contrast, the Cu­(II) and Zn­(II) analogues display well-defined π-holes, with *V_hole,mean_* values of 13.07 and 30.30 kcal mol^–1^, respectively, following the trend established in this work. The results are in good agreement with experimental observations, whereby {Ni­(cyclen)}^2+^ acts primarily as a counterion, while {Cu­(cyclen)}^2+^ and {Zn­(cyclen)}^2+^ exhibit directional binding interactions with the decavanadate. The *V_s_,_max_* descriptor is strongly positive for all {TM­(cyclen)}^2+^ fragments, as expected given their cationic nature. Considered in isolation, this could suggest the presence of a π-hole in every case; however, the Ni­(II) system clearly lacks this directional feature. In this context, *V*_*hole*,*mean*_ provides a more reliable descriptor of the localized electron depletion associated with a π-hole.

**Table 1:** π-Hole Descriptors for the {TM­(cyclen)}^2+^ Moieties with TM = Ni­(II), Cu­(II) and Zn­(II) Calculated for the Crystallographic Geometries Reported in the Literature^38^

{TM(cyclen)}^2+^	*V_s_,_max_*/kcal mol^–1^	*V*_*hole,mean*_/kcal mol^–1^	*V_hole,max_*/kcal mol^–1^	*V_hole,min_*/ kcal mol^–1^
{Ni(cyclen)}^2+^ ^a^	197.04	–0.90	1.90	–5.76
{Cu(cyclen)}^2+^	206.96	13.07	13.89	12.18
{Zn(cyclen)}^2+^	240.60	30.30	41.83	19.76

a Failed sectoral validation, *V*_*hole*_,_*mean*_ values reported for comparison.

### Cyclen Methyl and Ethyl Substitution Effects into π-Hole

The methyl and ethyl substitution patterns incorporated into the {TM­(cyclen)}^2+^ data set introduce a third, structural variable that modulates both the geometric accessibility and the electronic character of the axial region. Across the complexes analyzed, the identity and spatial orientation of the N-substituents exerted a nonmonotonical influence on π-hole presence. While the parent unsubstituted complexes exhibited pronounced axial electron depletion, the introduction of alkyl groups, particularly ethyl substituents, frequently attenuated, displaced, or entirely suppressed π-hole development. These effects arise from a combination of steric encumbrance and asymmetric polarization induced by uneven substitution patterns, and they constitute a key element of the observed variability in π-hole intensity. To bring light into this behavior, the substitution effects were analyzed using an approach that englobes conformational and steric effects.

The most direct influence of substitution is steric. Ethyl groups, owing to their larger size and rotatable N–C and C–C bonds, exhibit a much greater tendency than methyl groups to bend toward the axial region during semiempirical and DFT optimizations (Figure 8). When an ethyl group reorients in this manner, one or more of its C–H bonds projects into the space directly above the metal center, effectively occupying the region in which a π-hole would otherwise localize. These cases correspond to structures classified as π-hole–absent by the sectoral validation algorithm. Geometries in which an ethyl substituent intrudes within the π-hole region (the 1.0 Å cylinder used to define a π-hole candidate) generally did not show local ESP maxima. Underscoring that steric shielding by the substituent may prevent the formation of a meaningful positive potential above the N_4_-plane.

**Figure 8:**
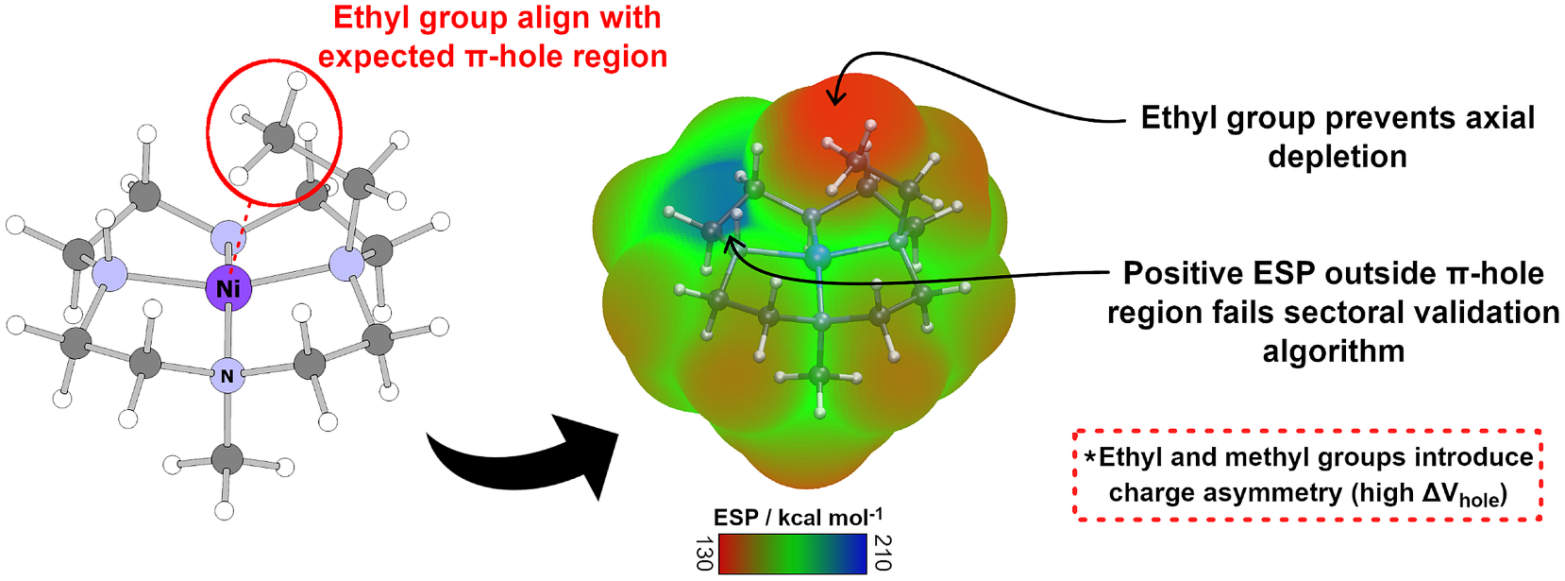
Structural and electrostatic origin of π-hole modulation in substituted cyclen complexes. Left: molecular structure highlighting alignment of an ethyl substituent with the axial region above the metal center. Right: electrostatic potential (ESP) mapped surface showing how the ethyl group perturbs the axial positive region, preventing depletion along the π-axis and introducing charge asymmetry. Positive ESP outside the canonical sector fails the sector-based π-hole validation criterion.

Beyond steric shielding, the electronic nature of the substituents subtly modulates π-hole depth through inductive donation and steric hindrances, impacting M–N bonding. Ethyl groups are slightly more electron-donating than methyl groups but also tend to create additional repulsion inside the macrocyclic ligand, which manifests as modest but systematic changes in average M–N distances. Across the data set, N-ethyl-substituted complexes exhibit mean M–N distances approximately 0.01–0.05 Å longer than their all-methyl or unsubstituted counterparts (Figure 9). Although this variation appears small, it plays a measurable role in redistributing electron density around the metal. Lengthening of the σ-donor M–N bonds weaken equatorial charge donation, thereby reducing metal-centered electron density and enhancing the polarization responsible for axial depletion. Under conditions where steric obstruction is maximized, ethyl substitution can therefore deepen the π-hole. Indeed, in conformers such as UUUU, where steric congestion is naturally heightened, complexes bearing multiple ethyl groups display some of the largest π-hole intensities observed for each metal ion.

**Figure 9:**
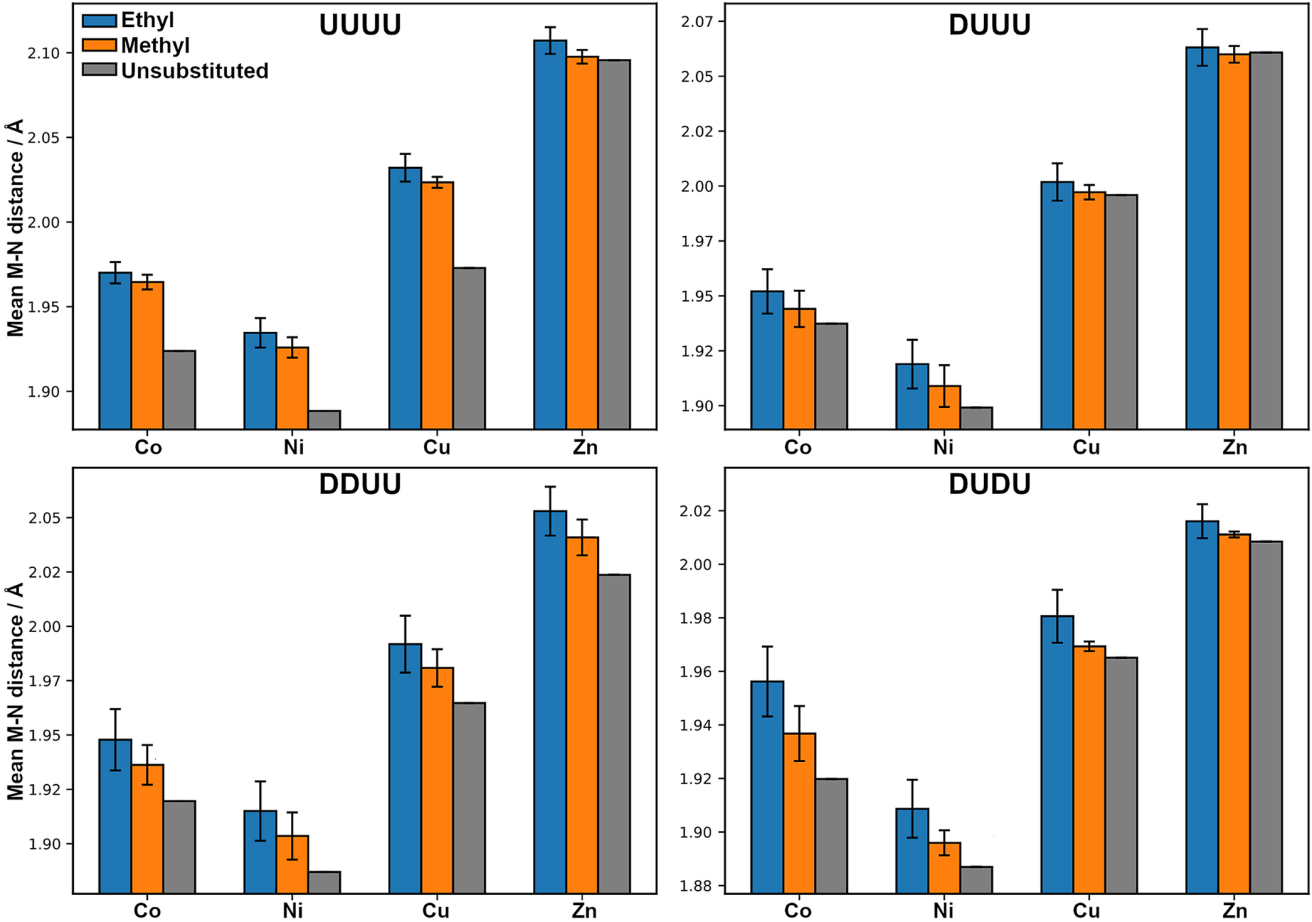
Mean metal–nitrogen bond distances for Co­(II), Ni­(II), Cu­(II), and Zn­(II) complexes across the four conformational patterns (UUUU, DUUU, DDUU, DUDU) and substitution levels (ethyl, methyl, and unsubstituted). Bars represent averages and error bars indicate standard deviations.

### π-Hole Asymmetry and Asymmetry Angle Case Study

To better visualize π-hole asymmetry, we conducted a case study comparing the methylation of Co­(II) structures in the UUUU conformer (Figure 10a), albeit it could be expanded to the other TM. This substituent topology was selected due to the presence of all R groups positioned above the N_4_-plane, making it possible to directly evaluate the impact of the substituents to Δ*V_hole_* values. It is also useful to introduce the angle between the sectors containing *V_hole,max_* and *V_hole,min_* (Δ*V_angle_*), as a practical way to define the direction of electron depletion asymmetry (Figure 10b–f).

**Figure 10:**
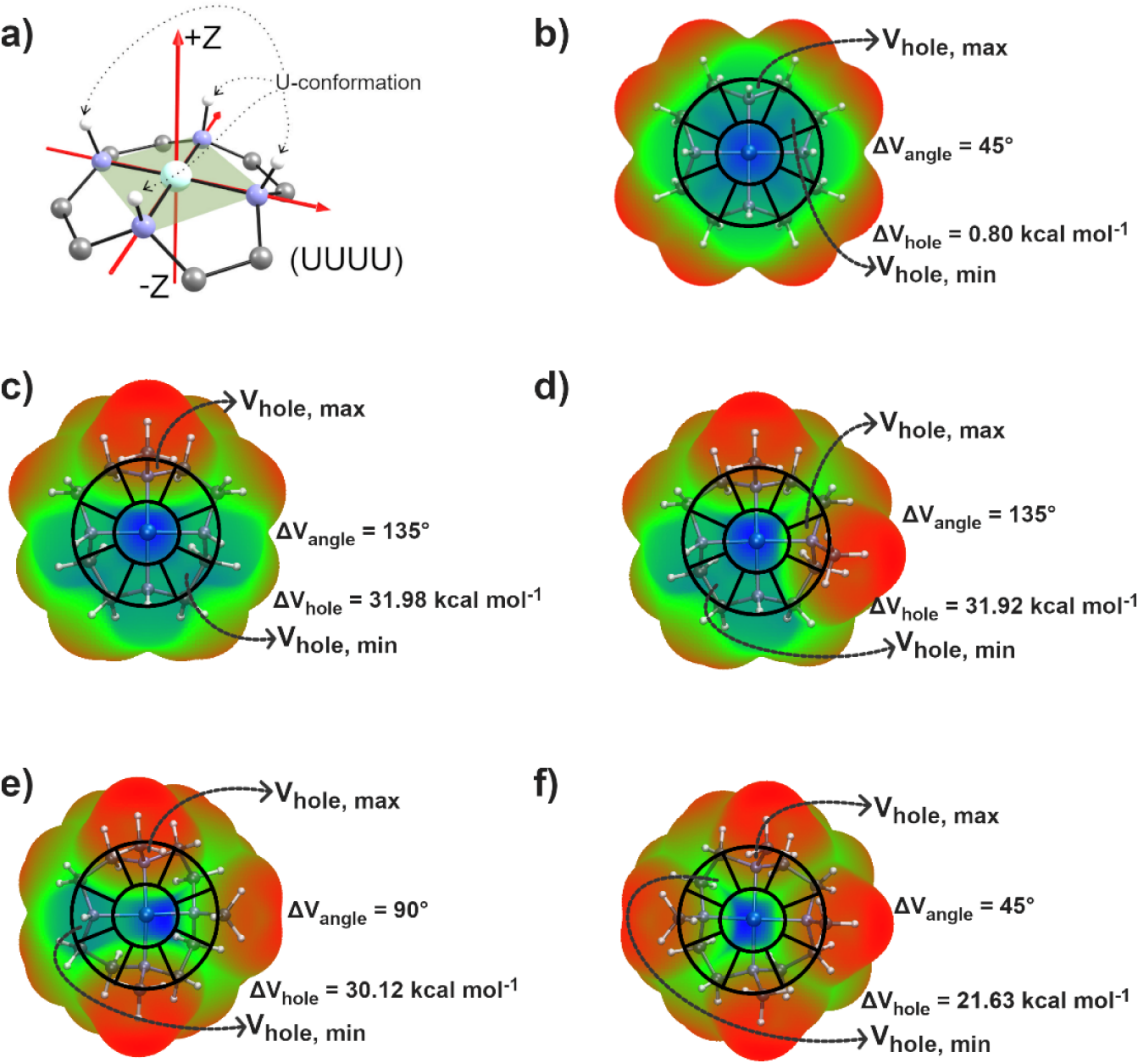
Analysis of the angular anisotropy of the metal-centered π-hole in the {TM­(cyclen)}^2+^ fragment. (a) Reference coordinate system used for the analysis, where the *Z* axis is perpendicular to the N_4_-plane in the UUUU conformation. (b–f) Molecular electrostatic potential (MEP) mapped on the electron density isosurface (*ρ* = 0.001 a.u.). The extrema of the electrostatic potential around the metal center define the π-hole anisotropy. The π-hole magnitude is quantified as Δ*V*_*hole*_ = Δ*V*_*hole*_,_*max*_ – *V_hole_,*_*min*_ where *V_hole_,*_*max*_ and *V*_*hole*,min_ correspond to the maximum and minimum potential depth sectors in the circular sampling path around the metal. The angular separation between the sectors containing the extrema is defined as Δ*V*_*angle*_. Blue indicates more positive electrostatic potential (π-hole region) and red indicates negative (or less positive) potential.

Taking {Co­(cyclen)}^2+^ as a starting point, the unsubstituted complex shows negligible asymmetry (Δ*V_hole_* = 0.80 kcal mol^–1^), as expected for a quasi-planar micrometry (Figure 10b). The substitution of one hydrogen by a methyl group creates a strong π-hole asymmetry, with a value of Δ*V_hole_* = 31.98 kcal mol^–1^ (Figure 10c). The subsequent clockwise addition of a second methyl group keeps the polarized distribution of electron density over the molecular plane (Δ*V_hole_* = 31.92 kcal mol^–1^) (Figure 10d). A third (Δ*V_hole_* = 30.12 kcal mol^–1^) and particularly a fourth (Δ*V_hole_* = 21.63 kcal mol^–1^) methyl group substitution tends to reduce the ESP asymmetry with the inclusion of electrostatic perturbations around the π-hole region (Figure 10e and f). Methyl groups, being compact, preserve axial accessibility and therefore yield ESP surfaces in which the algorithm consistently detects well-defined π-holes, while also revealing how local substituent fields introduce controllable asymmetry. This example demonstrates the importance of determining and analyzing an enlarged region around the π-hole to accurately describe its depth and asymmetry. In this context, N-substitution emerges in the algorithmic analysis as a bidirectional modulator: depending on its spatial disposition, it can either reinforce or attenuate axial electron depletion, altering both π-hole strength and the angular distribution used for asymmetry classification.

## Conclusion

This study establishes a high-throughput computational framework for identifying and quantifying metal-centered π-holes in pseudoplanar {TM­(cyclen)}^2+^ complexes. Using automated structure generation, xTB preoptimization, DFT calculations at the ωB97X-D3/def2-SVP level, and a sector-validated detection algorithm, 1296 complexes were analyzed, spanning four metals, four cyclen conformations, and all possible methyl/ethyl N-substitution patterns. Within this set, validated π-holes were found in 84.6% of the structures, showing that axial electrostatic depletion is a common feature of this ligand platform but is not invariant with respect to metal, conformation or substitution pattern. The descriptors used here show that *V_s_*,*_max_* alone is not sufficient to characterize π-hole behavior in charged transition-metal complexes, whereas the relative metrics derived from local sectoral comparisons provide a more robust measure of π-hole intensity and anisotropy. Also, results further show that the π-hole magnitude depends on the combined effects of electronic structure and geometry.

Across the series examined, the mean π-hole intensity follows the order Zn­(II) > Co­(II) > Cu­(II) > Ni­(II), in good agreement with experimental data observed in the literature.^14,38^ Conformational distortions that increase tetrahedral character generally reduce π-hole intensity, whereas off-plane distortions associated with the UUUU arrangement tend to increase it. These trends are consistent with changes in equatorial σ-donation and in the occupation and energetic disposition of metal-centered orbitals that control electron density along the axis normal to the N_4_-plane. N-substitution introduces an additional and non-negligible moduling element. Methyl substituents usually preserve axial accessibility while modulating the angular distribution of the electrostatic potential. Ethyl substituents exhibits a stronger structural effect because their conformational flexibility can orient C–H bonds in the *z*-axis region, suppressing local maxima and preventing π-hole validation in otherwise favorable electronic environments. When this steric blocking is absent, substitution can also increase π-hole intensity by lengthening M–N bonds and weakening equatorial donation.

Taken together, the results discussed above provide a quantitative framework for evaluating metal-centered π-holes in coordination complexes. Rather than relying solely on qualitative inspection of electrostatic potential maps or on absolute *V_s,max_* values, the sector-based metrics introduced here allow the π-hole to be characterized through measurable quantities such as its mean depth (*V_hole_*,*_mean_*), its asymmetry value (Δ*V*_*hole*_) and its angular anisotropy (Δ*V*_*angle*_). These descriptors capture both the magnitude and spatial distribution of the electrostatic depletion above the metal center, enabling direct comparison across metals, conformations, and substitution patterns. By defining π-hole intensity in terms of these quantitative parameters, this approach establishes a reproducible basis for assessing axial electrophilicity in transition-metal complexes and provides a transferable protocol for analyzing electrostatic anisotropy in other environments.

## Supplementary Material



## Data Availability

Computational details, data, and results that complement the study presented herein are available in the Supporting Information at http://www.xxxxxxxxxx. The softwares developed to conduct the study are available upon author request along with the curated data set obtained. All the optimized structures and outputs are available at NOMAD (https://doi.org/10.17172/nomad.vw6z-b7wn).

## Notes

Complementary graphical representation of cyclen conformations, schematic representation of molecular alignment procedure, grid-sensitivity analysis of π-hole and Δ*V*_*hole*_ descriptors, mean computational run times for the Ni­(II), Co­(II), Cu­(II), and Zn­(II) data sets, mean geometries of the {TM­(cyclen)}^2+^ complexes, comparison of electrostatic descriptors obtained using def2-SVP and def2-TZVP basis set and structures of {TM­(cyclen)}^2+^ complexes interacting with the decavanadate anion (PDF)

## Abbreviation

CC BY: Creative Commons Attribution license
DFT: Density Functional Theory
ESP: Electrostatic Surface Potential
MEP: Molecular Electrostatic Potential
TM: Transition Metal
MO: Molecular Orbital
Cyclen: 1,4,7,10-tetraazacyclododecane
N_4_-plane: Plane defined by the four nitrogen atoms of cyclen
RMSD: Root Mean Squared Deviation
UUUU, DUUU, DUDU, DDUU: Cyclen conformers defined by up/down orientation of N-substituents relative to the N_4_-plane
ρ: Electron density
V: Electrostatic potential
∂V/∂r: First derivative of the electrostatic potential
∂^2^V/∂r^2^: Second derivative of the electrostatic potential
*V_s,max_*: Maximum value of electrostatic potential
*V_hole,max_*: Maximum π-hole potential relative to sector background
*V_hole,min_*: Minimum π-hole potential relative to sector background
*V_hole,mean_*: Mean π-hole potential relative to sector background
Δ*V*_*hole*_: π-hole asymmetry descriptor based on *V_hole,min_* and *V_hole,max_*
Δ*V_angle_*: Angular separation between sectors defining π-hole asymmetry­(Δ*V*_hole_)
τ_4_: Four-coordinate geometry index describing distortion between square-planar and tetrahedral geometries
