# Supplementary material for: Quantitative Analysis of Metal-Centered π‑Holes in {TM(cyclen)}2+ Complexes
Source: Inorg Chem. 2026 May 11;65(20):11378–91. doi: 10.1021/acs.inorgchem.6c01328 (PMC13213900; doi:10.1021/acs.inorgchem.6c01328)
Supplement: Supplementary file 1 [file ic6c01328_si_001.pdf]

# Supporting Information

## Quantitative Analysis of Metal-Centered π-Holes in $\{TM(cyclen)\}^{2+}$ Complexes

Lucas Gian Fachini[#], Heloísa de Souza Camilo[#], Matteo Briganti[&], Eduardo Lemos de Sá[#], Giovana Gioppo Nunes[#*]

[#] Departamento de Química, Universidade Federal do Paraná, Centro Politécnico, Jardim das Américas, 81530-900, Curitiba, PR, Brazil.

[&] Dipartimento di Chimica Ugo Schiff and INSTM RU, Università degli Studii Firenze, Via della Lastruccia 3–13, 50019, Sesto Fiorentino, FI, Italy.

Corresponding author: Giovana Gioppo Nunes, e-mail: nunesgg@ufpr.br

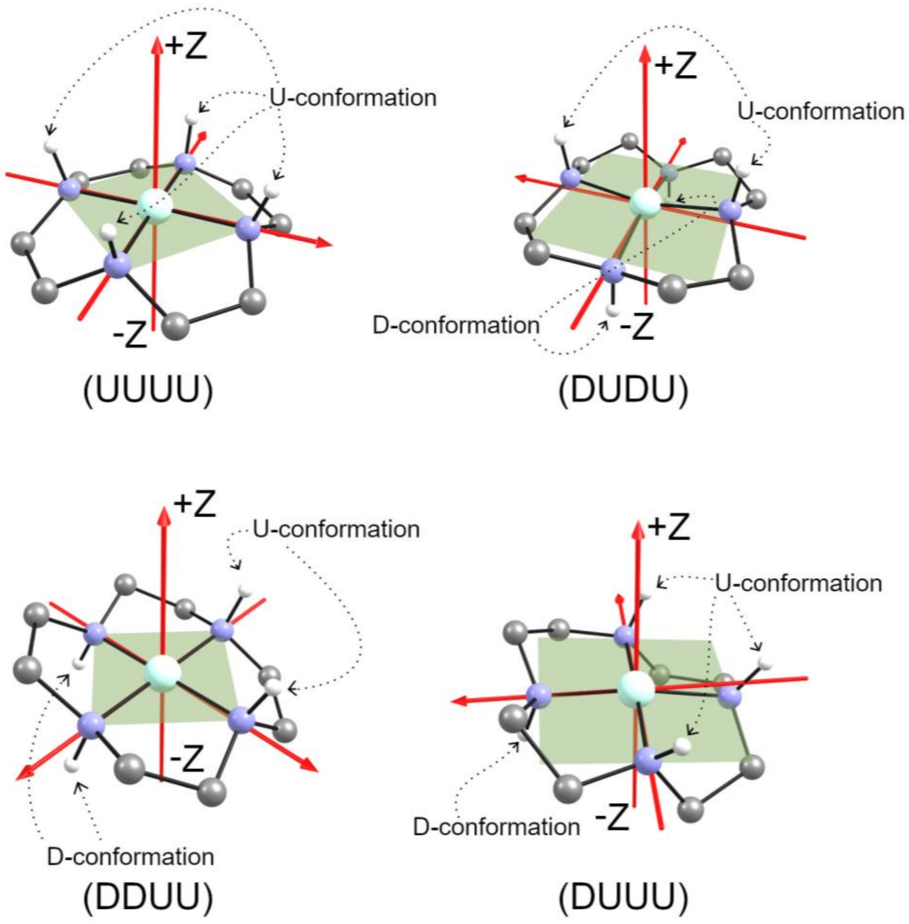


Figure S1. Graphical representation of the U/D notation used to describe cyclen conformations in $\{TM(cyclen)\}^{2+}$ complexes. The four nitrogen atoms define the $N_4$ mean plane (light green) and its normal vector (z-axis). An N–R vector pointing above the plane is labeled U (up), whereas one pointing below the plane is labeled D (down). Different U/D combinations generate the UUUU, DUUU, DUDU, and DDUU conformers analyzed in this work.

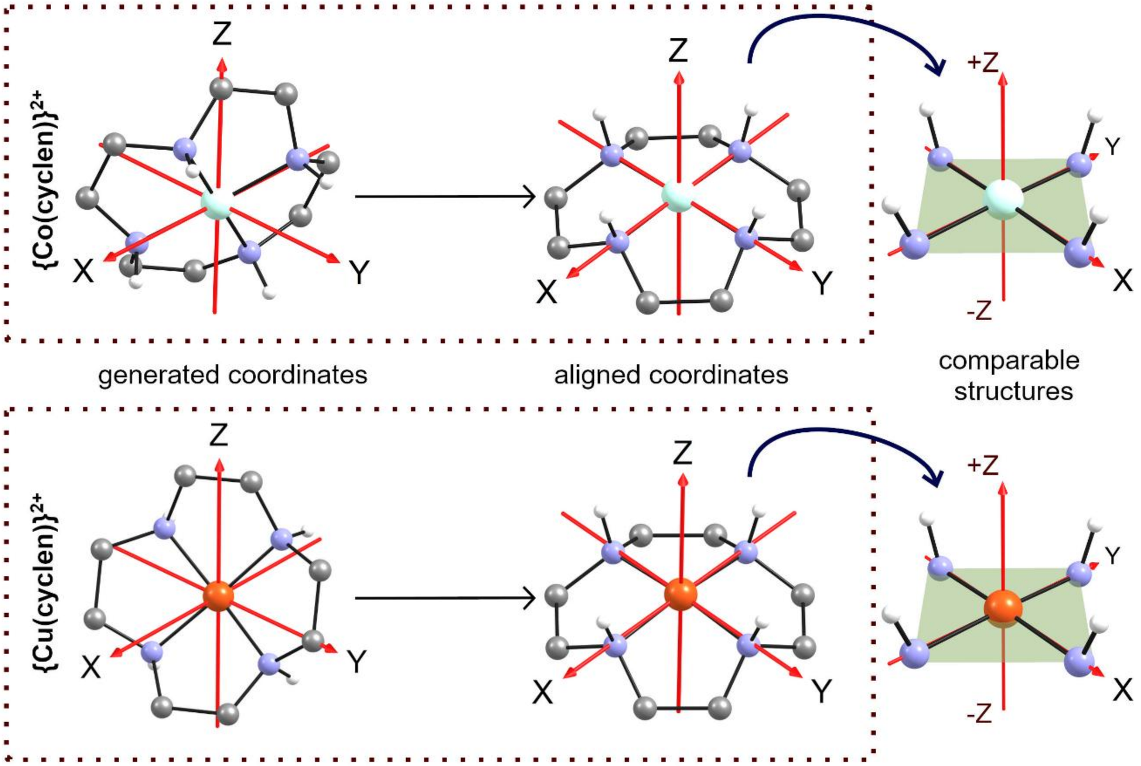


Figure S2. Schematic representation of the molecular alignment procedure applied to all conformers prior to ESP analysis. The $N_4$ coordination plane defines the molecular frame, with its centroid as the origin, the plane normal (n̂) as the pseudo-z axis, and in-plane x and y axes defined from the shortest non-collinear N–N vectors.

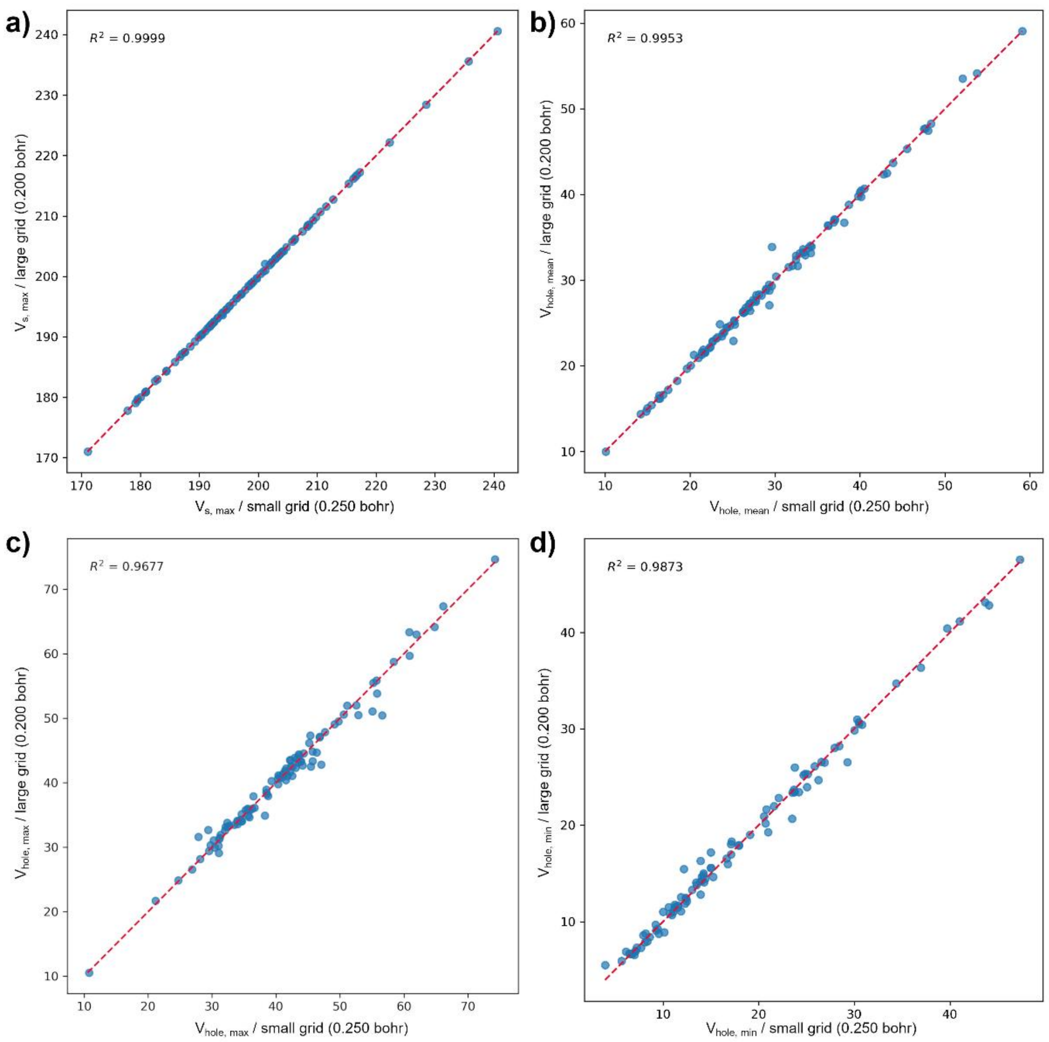


Figure S3. Grid-sensitivity analysis of π-hole descriptors obtained with grid spacings of 0.25 and 0.20 bohr for a representative subset of 100 complexes. Correlation plots compare values computed with the two grids for (a)$V_{s,\ max}$, (b)$V_{hole,\ mean}$, (c)$V_{hole,\ max}$, and (d)$V_{hole,\ min}$. The dashed red lines indicate ideal agreement. High linear correlations and small deviations confirm the weak dependence of these descriptors on grid resolution.

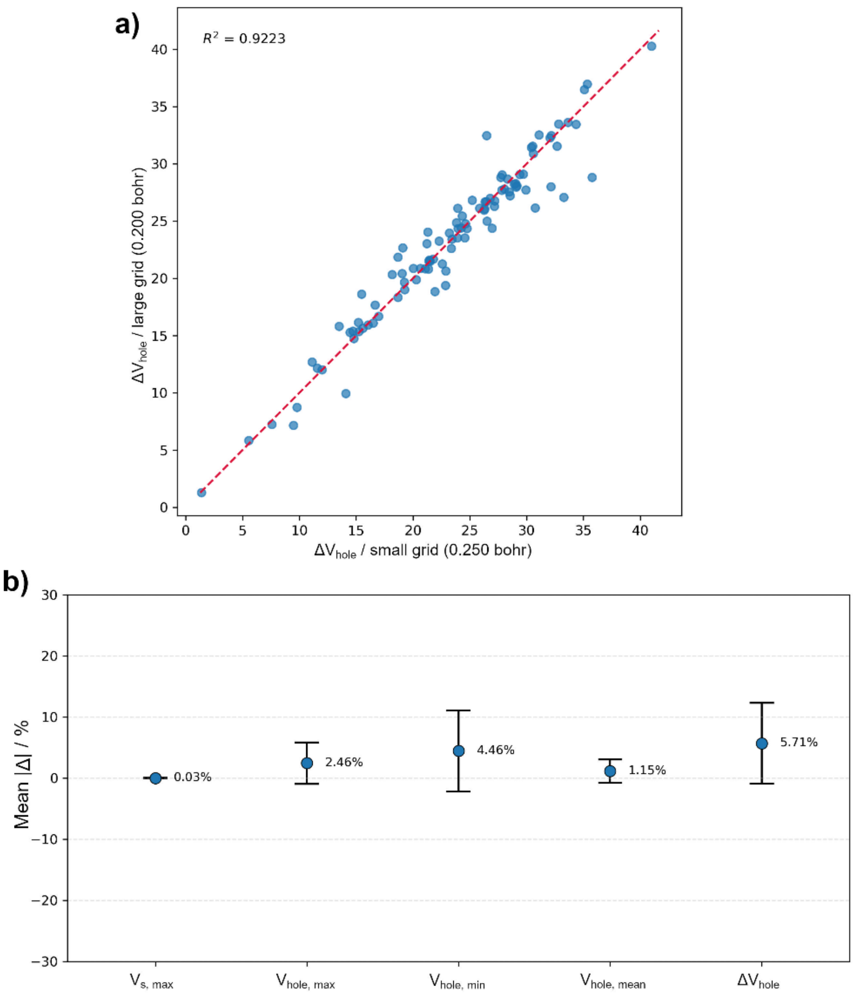


Figure S4. a) Grid-sensitivity analysis of the $\Delta V_{hole}$ descriptor computed with grid spacings of 0.25 and 0.20 bohr for the same subset of 100 complexes. The correlation plot compares values obtained with the two grid resolutions. The dashed red line represents ideal agreement. b) Mean relative deviations (in %) of all π-hole descriptors evaluated in the grid-sensitivity tests, including those shown in Fig. S3 and panel (a), summarizing the overall grid dependence across descriptors.

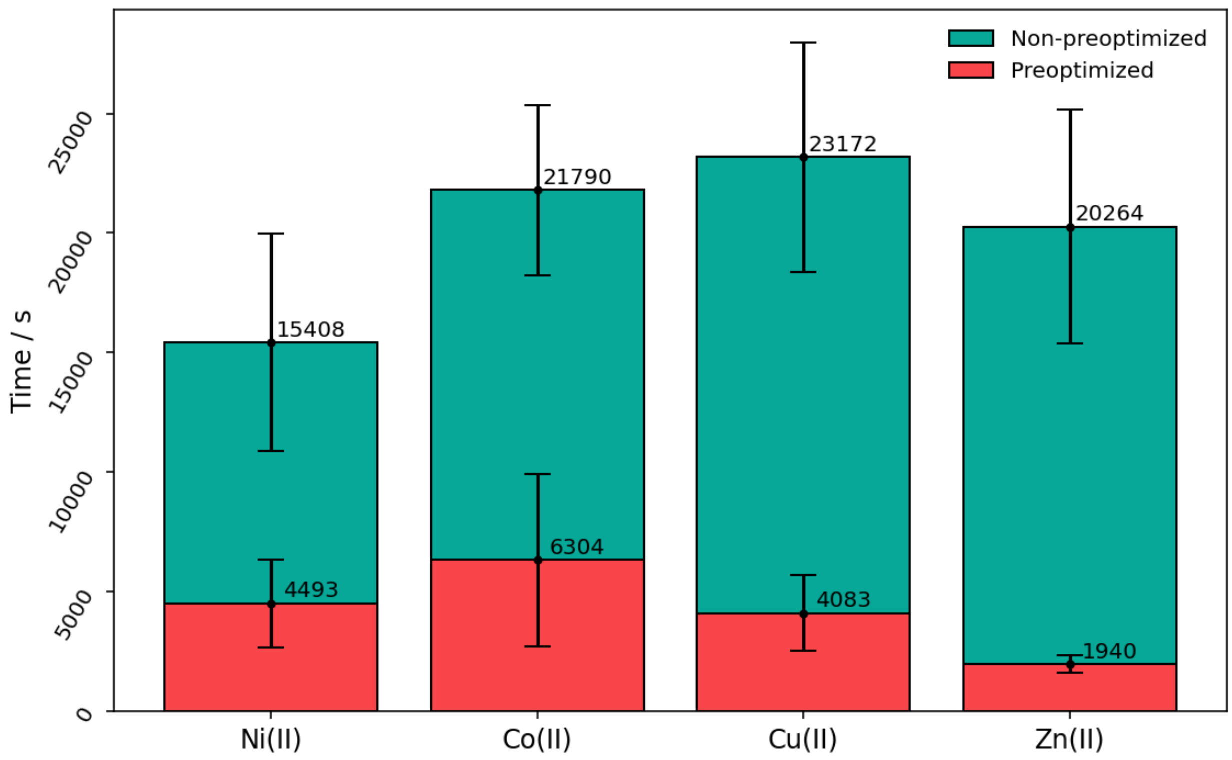


Figure S5. Mean computational run times for the Ni(II), Co(II), Cu(II), and Zn(II) datasets (10 compounds for each metal) comparing non-preoptimized structures (green) and preoptimized structures (red). Bars represent average total run time in seconds, and error bars correspond to the standard deviation across all calculations in each set. Numerical labels indicate the mean values for each condition, highlighting the substantial reduction in computational cost achieved through preoptimization. The pre-optimization was conducted using the GFN2-xTB semi-empirical Hamiltonian.

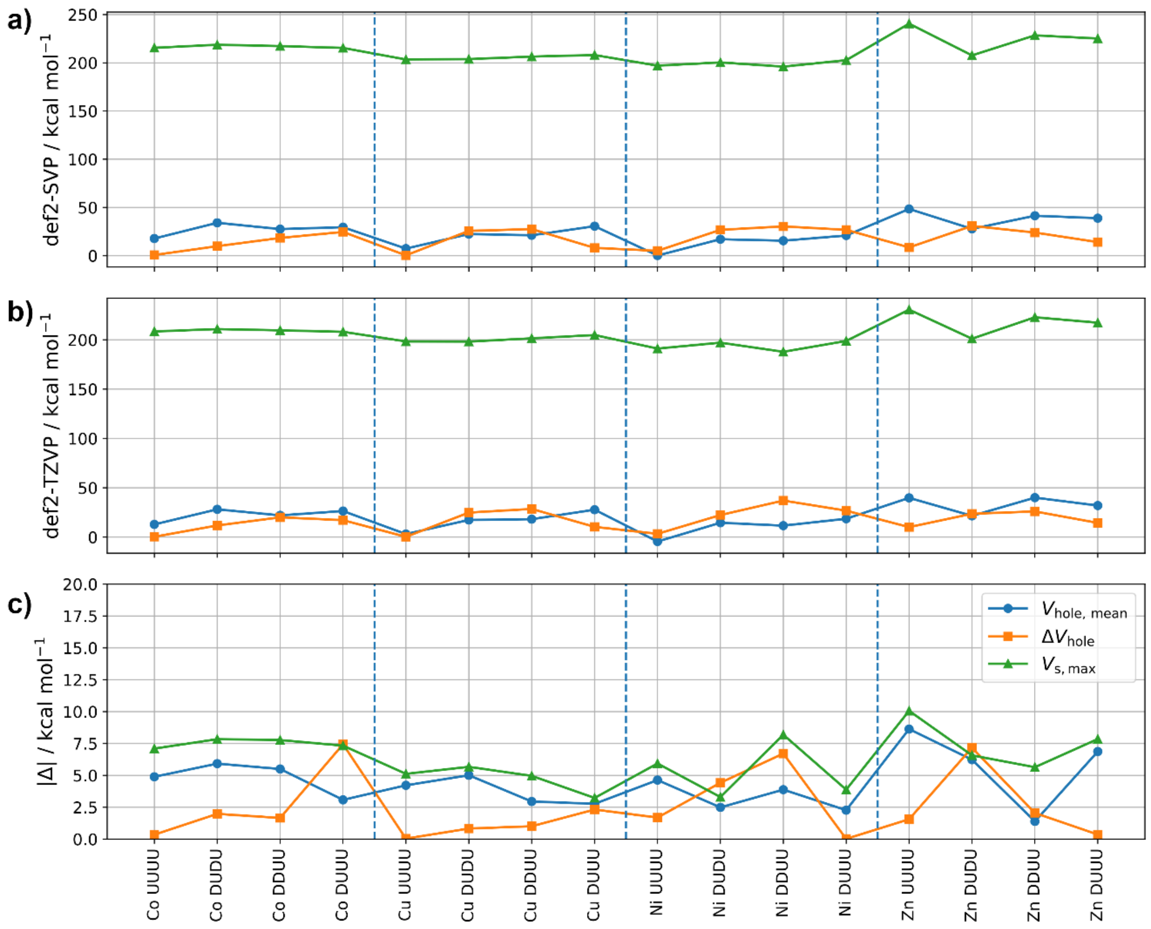


Figure S6. Comparison of electrostatic descriptors obtained using the def2-SVP and def2-TZVP basis sets across representative $\{TM(cyclen)\}^{2+}$ complexes. (a) Absolute values of $V_{\text{hole,mean}}$, $\Delta V_{\text{hole}}$, and $V_{s,\text{max}}$ computed at the def2-SVP level. (b) Corresponding values obtained with the def2-TZVP basis set. (c) Absolute deviations | Δ |between the two basis sets for each descriptor. Vertical dashed lines separate different metal centers (Co, Cu, Ni, Zn), while individual points correspond to distinct macrocyclic conformations.

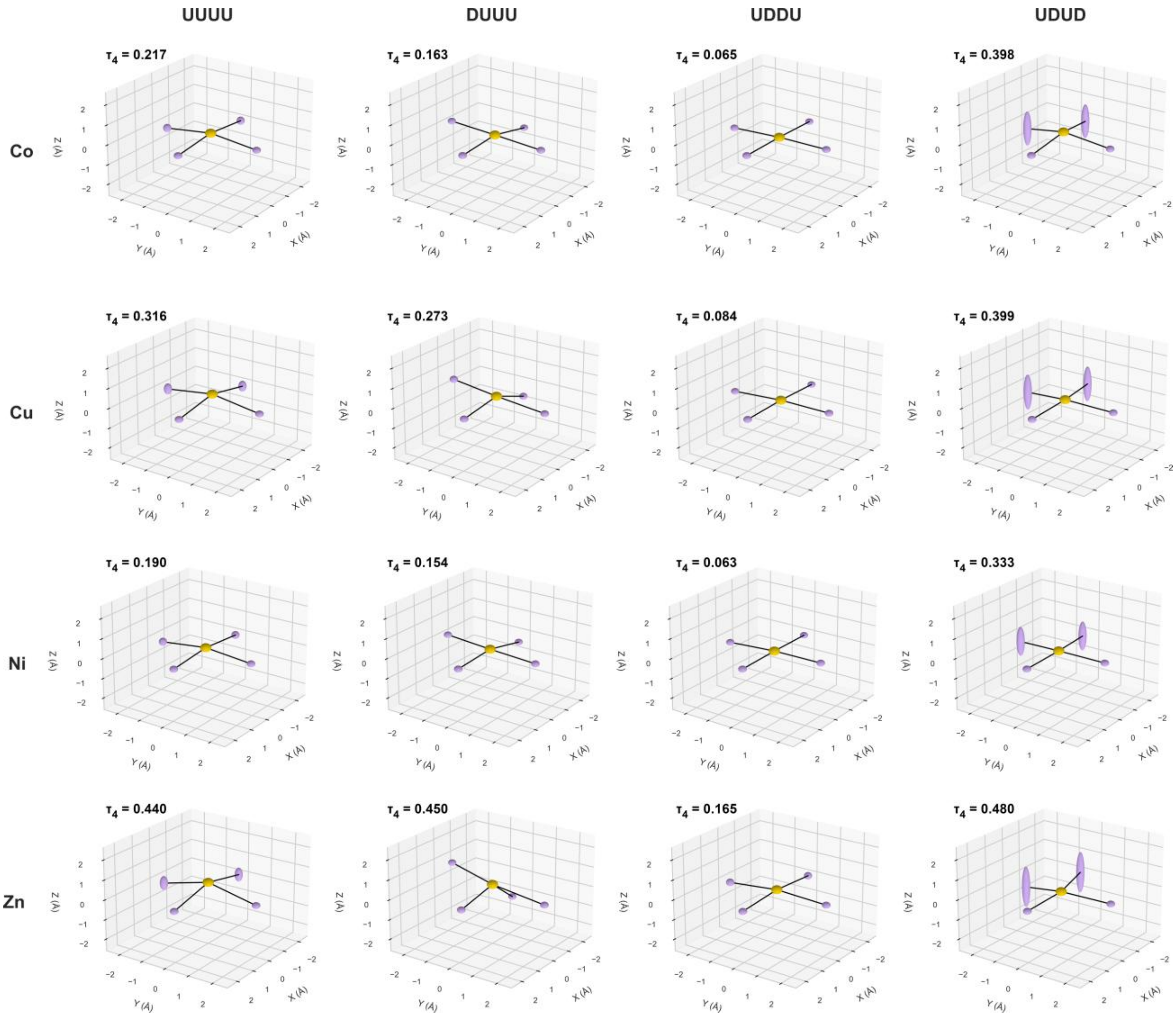


Figure S7. Mean geometries of the $\{TM(cyclen)\}^{2+}$ complexes for the four macrocyclic conformations (UUUU, DUUU, DDDU, and UDUD) and the four transition metals investigated (Co(II), Cu(II), Ni(II), and Zn(II)). The $\tau_4$ geometry index is reported for each mean structure to indicate the degree of distortion from an ideal square-planar coordination environment. Purple ellipsoids represent the positional distributions of the nitrogen atoms across the dataset, constructed from the standard deviation of their coordinates, and illustrate how conformational distortions affect the spatial arrangement of the N donors around the metal center.

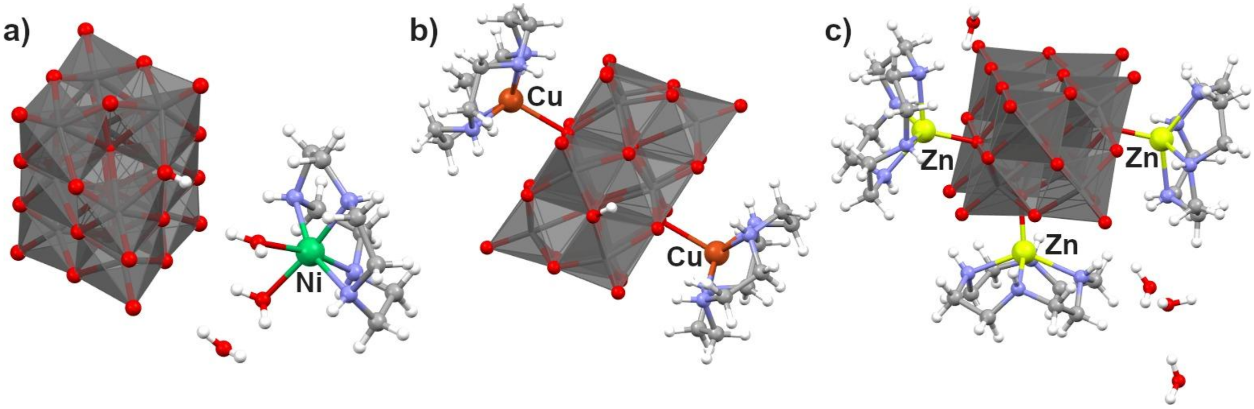


Figure S8. Schematic representation for structures of the ionic pair $[Ni(cyclen)(H_2O)_2]_2[H_2V_{10}O_{28}]\cdot 2H_2O$ and the discrete molecular entities $[\{Cu(cyclen)\}_2(H_2V_{10}O_{28})]\cdot 9H_2O$, and $[\{Zn(cyclen)\}_3(V_{10}O_{28})]\cdot 4H_2O$. Colors: zinc = yellow, nickel = green, copper = orange, nitrogen = blue, oxygen = red, hydrogen = white, carbon = gray. Vanadium atoms are represented as dark gray polyhedra.
